# Synergistic HER2-scFv mediated immunotherapy with targeted gemcitabine delivery harnessing biomimetic composite nanoparticles for enhanced metastatic tumor therapy

**DOI:** 10.1016/j.mtbio.2025.101784

**Published:** 2025-04-22

**Authors:** Xinan Wang, Wenhui Chu, Fuyu Du, Hongyu Fan, Zixuan Ye, Yaru Xue, Xianghan Zhang, Peng Yang, Yuqiong Xia, Zhihui Chen, Pengbo Ning

**Affiliations:** a School of Life Science and Technology, Xidian University, Xi'an, 710071, Shaanxi, PR China; b Engineering Research Center of Molecular & Neuroimaging, Ministry of Education, Xi'an, 710071, Shaanxi, PR China; c Department of Gastrointestinal Surgery Center, The First Affiliated Hospital, Sun Yat-sen University, Guangzhou, 510080, PR China; d Department of General Surgery, Guangxi Hospital Division of the First Affiliated Hospital, Sun Yat-sen University, Nanning, 530022, Guangxi, PR China

**Keywords:** Biomimetic nanoplatform, Anti-HER2, Macrophage membrane camouflage, Combined therapy, Metastatic tumors

## Abstract

Metastatic breast cancer poses a formidable challenge to global female health and public hygiene. Patients with HER2^+^ breast cancer typically demonstrate higher mortality rates and rapid recurrences. Despite the notable advantages in high effectiveness and precision for HER2^+^ cancer therapy, ADCs (Antibody-Drug Conjugates) still face limitations in rapid metabolism and off-target toxicity *invivo*. Recently, biomimetic cell membranes camouflaged nanocarriers exhibits remarkable capabilities in immune escape, extended drug circulation in the bloodstream, and enhanced targeted accumulation at tumor sites. In response, PLGA nanoparticles with engineered modified macrophage membrane coating was designed to develop a novel gene reprogramming biomimetic drug-loading nanoplatform. The nanoplatform enabled anti-HER2 antibodies to be stably expressed on the macrophage membrane (HM), making it more effective against HER2^+^ tumor cells, achieving precise targeted delivery of near-infrared (NIR) fluorescent dye IR780 or chemotherapy drugs GEM. When compared to nanoparticles coated with traditional macrophage membranes, GEM@PLGA@HM demonstrated outstanding HER2-targeting proficiency both *invitro* and *invivo*. Imaging studies conducted on small animals further verified the precise accumulation of GEM@PLGA@HM specifically within HER2^+^ tumors. When combined with HER2 antibodies, GEM treatment exhibited a marked improvement in antitumor effects, both *invitro* and *invivo*. Notably, the combined therapy demonstrated a significant 14-fold increase in cytotoxicity against HER2^+^ metastatic cancer cells (SK-OV-3) in comparison to GEM used alone, which was further proved mechanistically that GEM@PLGA@HM operated synergistically to hinder the progression and metastasis of HER2^+^ tumors by triggering apoptosis and concurrently inhibiting the PI3K/AKT and MAPK signaling cascades. This biomimetic nanoplatform represents a powerful tool for targeted chemotherapeutic delivery and holds great promise for the treatment of HER2^+^ primary and metastatic breast cancer.

## Highlights

• A targeted Antibody-Drug Membrane-Camouflaged Composite (ADMC) with HER2-scFv-engineered macrophage membrane camouflage was designed for metastatic tumor therapy.
• The ADMC nanoplatform targeted HER2 to deliver gemcitabine and boosted monotherapy's anti-tumor effects *in vitro* and *in vivo*.
• The ADMC nanoplatform inhibited HER2^+^ tumor growth and metastasis by inducing apoptosis and blocking PI3K/AKT and MAPK pathways.

## Graphical abstract

**Figure:**
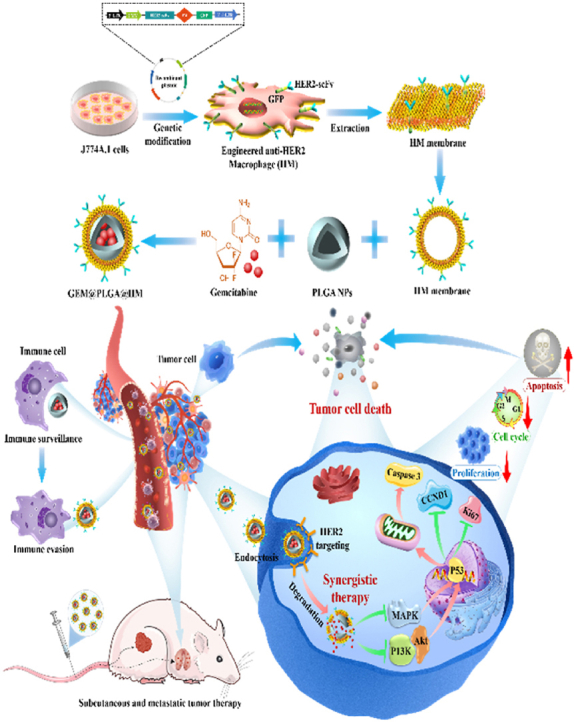

## 1 Introduction

Breast cancer stands as a prominent cause of morbidity and mortality among women globally, with an estimated 310,720 new cases, accounting for 32% of all cancer diagnoses in the United States in 2024 [1]. It is reported that between 20% and 30% of breast cancer patients ultimately progress to develop metastatic disease following their initial diagnosis and treatment [2]. According to the latest update from the American Cancer Society on breast cancer statistics, approximately 84% of invasive breast cancer cases in the U.S. are detected in women aged 50 and older, with 91% of diagnosed patients ultimately facing death from the disease [3]. Metastasis of cancer is one of the major challenges for the survival and treatment success of cancer patients. Overexpression of the human epidermal growth factor receptor 2 (HER2) gene can trigger uncontrolled cellular proliferation, ultimately leading to the development of tumors. Breast cancer patients with HER2 overexpression typically demonstrate reduced survival rates and accelerated recurrence rates [4]. Consequently, HER2 has emerged as a pivotal target in the treatment of breast cancer. Therapeutic strategies targeting HER2, including monoclonal antibody therapy, have become significant breakthroughs in the field of breast cancer therapy. For instance, the monoclonal antibody trastuzumab inhibits HER2 homodimerization by binding to the extracellular domain of HER2 to prevent its activation, leading to the antibody-dependent cytotoxicity of HER2^+^ cancer cells [5]. Despite the remarkable achievements of anti-HER2 monoclonal antibody therapy, drug resistance and side effects caused by off-target effects on normal tissues remain major challenges in HER2^+^ breast cancer therapy. Therefore, the combination of anti-HER2 targeted therapy with other therapeutic strategies like chemotherapy has been an urgent need to achieve better therapeutic efficacy of HER2^+^ breast cancer.

Compared to the limited therapeutic efficacy and reduced lethality towards tumor cells when monoclonal antibodies are used as a monotherapy, antibody-drug conjugate (ADC) is based on tumor-targeting monoclonal antibodies, which can be tightly bound to cytotoxic drugs through elaborately designed ligands, achieving exceptional targeting accuracy while ensuring a potent tumoricidal effect [6,7]. The ideal ADC should remain stable in the blood circulation while precisely targeting tumors and releasing the drug [8]. With the continuous expansion of targets, ADC technology is expected to gradually replace traditional chemotherapy methods in the future [7]. For instance, T-DM1, an anti-HER2 antibody drug conjugate, has been approved by the FDA and the European Medicines Agency (EMA) for adjuvant therapy in patients with HER2-positive metastatic disease, who have previously undergone treatment with trastuzumab and taxane [6]. SYD985 is a novel HER2-targeted ADC featuring a trastuzumab-like HER2 antibody coupled to the alkylate duocarmycin via a cleavable ligand. Currently, the TULIP Phase III clinical trial is investigating SYD985 in combination with trastuzumab for the treatment of metastatic HER2^+^ breast cancer [5,6]. While progress has been made in the fight against benign solid tumors, the development and application of ADCs have been facing numerous challenges, including rapid blood circulation, complex pharmacokinetics, inadequate drug release, and drug resistance, which severely impair their efficacy against metastatic tumor [9]. Additionally, in actual ADC therapy, premature release of cytotoxic drugs into the bloodstream can lead to severe hematotoxicity, hepatotoxicity and gastrointestinal reactions, mirroring the toxic side effects of traditional chemotherapy drugs [9]. The antibody component of ADCs may also elicit an immune response, leading to the secondary damage and ultimately nephrotoxicity [10].

Drawing inspiration from natural biological systems, the biomimetic nano-drug delivery system mediated by cell membranes has opened up new prospects and methodologies for precision medicine in oncology [11]. This revolutionary approach holds the potential to remedy the constraints of conventional ADCs system, effectively mitigating adverse reactions and negative toxicities while optimizing therapeutic efficacy. Notably, the macrophage membrane coating significantly prolonged the blood circulation time of these hybrid NGs and enabled them to traverse the blood-brain barrier (BBB), facilitating precise targeted delivery of chemotherapy and chemodynamic therapy (CDT) for orthotopic glioma [12]. Nonetheless, the application of native, unmodified macrophage membrane continues to pose considerable challenges in achieving precise drug targeting. In light of the specific target HER2 antigen in breast cancer, we have undertaken a rigorous process of functional engineering and modification of macrophage membranes. Drawing inspiration from the intricate mechanisms of viral particle assembly and membrane budding, gene reprogramming technology was applied to employ lentiviral vectors for the recombinant expression of anti-HER2 antibodies on the surface of macrophage membranes, followed by the extraction of the engineered modified macrophage membranes. Compared to direct physicochemical modification of macrophage membranes via adsorption or covalent binding, this approach provides substantial benefits by avoiding pitfalls such as inefficient coupling, diminished targeting precision and efficacy resulting from antibody random orientation, and the risk of chemical toxicity.

**Scheme 1:**
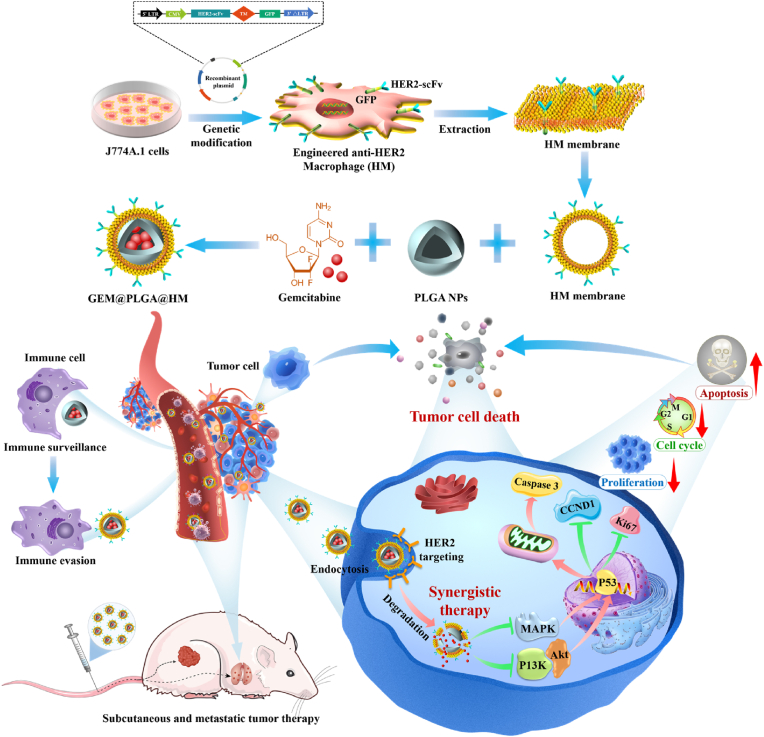
Illustration for the synergistic anti-HER2^+^ tumor effects of functionalized macrophage-encapsulated PLGA nanoparticles (GEM@PLGA@HM) via combining HER2 targeted therapy and chemotherapy.

In the present study, the novel anti-HER2 macrophage (Anti-HER2 Macrophage, HM)integrates the active targeting capability towards HER2-positive tumors with the therapeutic efficacy of monoclonal antibodies, marking a pioneering approach to eliminate tumor cells. Additionally, poly (lactic-co-glycolic acid) (PLGA) NPs loaded with chemotherapy drug Gemcitabine (GEM) was synthesized. This Antibody-Drug Membrane-Camouflaged Composite (ADMC) was employed to develop a macrophage membrane-mediated bionic drug delivery system, termed GEM@PLGA@HM. This innovative system represents a promising strategy to substantially elevate the accuracy of gemcitabine delivery to tumor sites and augment the therapeutic efficacy for solid tumors, particularly those of metastatic nature. Consequently, precise combination therapy can be achieved. By merging these two strategies, we successfully formulated HER2 antibody-modified engineered macrophage membrane-coated GEM-loaded nanoparticles (GEM@PLGA@HM), which exhibit enhanced targeting efficiency and a robust combined therapeutic effect (Scheme 1). A battery of experiments was conducted to characterize the properties of GEM@PLGA@HM, focusing on its immune escape capabilities and, notably, its targeting proficiency in tumor cells and tumor-bearing mice. Furthermore, the therapeutic efficacy of GEM@PLGA@HM at the cellular level was validated to assess the combination therapy's effectiveness and delve into the underlying molecular mechanisms. Ultimately, mouse models of solid and metastatic tumors were established for treatment, aiming to investigate whether GEM@PLGA@HM could enhance therapeutic efficacy while ensuring biosafety. Through these rigorous validations, we aimed to evaluate whether GEM@PLGA@HM achieves a precise and effective combined treatment outcome for breast cancer, particularly metastatic breast cancer. Remarkably, functionalized macrophage membranes showcase the advantages of personalized cancer treatment, as the extracellular antibody domains specifically bind to HER2 receptors to target and eliminate HER2^+^ breast cancer cells, while facilitating efficient drug delivery. The integration mode of our biomimetic nanoplatform stands in stark contrast to antibody-drug conjugates (ADCs). Compared to ADCs, GEM@PLGA@HM minimizes systemic toxicity and side effects, offering intrinsic tumor enrichment tendencies and inherent biological therapeutic functions for effective targeting and enhanced combination therapy of tumors.

## 2 Materials and methods

### 2.1 Construction and characterization of anti-HER2 macrophages

The sequence for HER2-scFv-GFP was sourced from the National Center for Biotechnology Information and synthesized by Shanghai Generay Biotech Co., Ltd. Meanwhile, the vector pCDH-CMV-MCS-EF1-Puro was acquired from YouBio Co., Ltd. To proceed with lentiviral packaging, a recombinant plasmid harboring the desired DNA fragment was constructed through enzymatic digestion, adhering to protocols outlined in prior publication [13].

In order to construct macrophages with stable expression of anti-HER2 antibodies on the cell membrane, J774A.1 cells were further genetically modified to achieve precise targeting of HER2^+^ cancer cells. In the process of transduction, lentivirus filtrates obtained from the above steps were added to the culture medium to infect J774A.1 cells, so that the HER2-scFv gene could be stably expressed on the cell membrane. After 24 h, the culture medium was changed to DMEM complete culture medium for a 48-h continued cultivation. Subsequently, puromycin was used for screening and for further expanded culture, thus macrophages expressing HER2-scFV were successfully constructed. Laser scanning confocal microscope observation was performed and macrophage RNA was extracted for real-time fluorescent quantitative PCR to determine the lentiviral infection. To further confirm the expression of HER2-scFv and evaluate its binding efficacy to HER2 protein on the cell membrane, the aforementioned stably transfected J774A.1 cells were incubated with 100 μL of 1 mg/mL PE-labeled HER2 protein for a duration of 4–6 h. Following meticulous washing and sample preparation, the fluorescence intensity of PE (with an emission wavelength of 575 ± 25 nm) was measured using flow cytometry.

### 2.2 GEM@PLGA preparation and synthesis optimization

GEM@PLGA was synthesized via a double emulsification-evaporation technique. Initially, PEG-PLGA was dissolved in 1 mL of dichloromethane (DCM). Subsequently, a GEM solution was carefully introduced to this mixture, which was then emulsified for 1 min using an ultrasonic device set to a pulsed mode (9 s on, 3 s off, at 50 W power). A 5% (w/v) aqueous solution of polyvinyl alcohol (PVA) was gradually added to the primary emulsion, followed by the addition of a 0.5% PVA solution. Further ultrasonic treatment, lasting 15 min, was applied to create a water-in-oil-in-water (w/o/w) double emulsion. After evaporating the w/o/w emulsion using a rotary evaporator for 20 min, vacuum distillation was employed to remove the organic solvent. During the synthesis of the nanoparticles, the ultrasonic intensity was varied to 50 W, 75 W, and 100 W to investigate how different intensities, both before and after the addition of PVA, influenced the properties of the nanoparticles. Finally, the size and zeta potential of the nanoparticles were characterized using a Zetasizer Nano-ZS instrument from Malvern Instruments (UK).

To determine the drug loading rate of GEM@PLGA, the nanoparticles were first dispersed in deionized water, followed by adding acetonitrile and ultrasonic treatment for 1 h, which helps to release the GEM from the nanoparticles into the supernatant. After ultrasonic demulsification, the mixture was centrifuged for 15 min and the supernatant was collected. The GEM content in the supernatant was determined by ultraviolet spectrophotometer. The calculation formula of drug loading rate is as follows: Drug loading rate (%) = GEM loading quantity/GEM feeding quantity × 100%.

### 2.3 Cell culture

Mouse breast cancer cell line 4T1 and human ovarian adenocarcinoma cell line SK-OV-3, both HER2-positive, were propagated in RPMI-1640 medium (Hyclone) containing 10% fetal bovine serum (FBS; GIBCO) and 1% penicillin/streptomycin. These cells were maintained in an incubator at 37°C with an atmosphere composed of 95% air and 5% CO_2_. Additionally, human embryonic kidney cell line 293T and mouse macrophage cell line J774A.1 were cultivated in Dulbecco's modified Eagle's medium (DMEM), enriched with 10% FBS, 100 U/mL of penicillin, and 100 μg/mL of streptomycin. These cultures were maintained at 37°C in an environment with 5% CO_2_.

### 2.4 Isolation of engineered macrophage membrane

Following the process of collection and centrifugation, the engineered macrophages were resuspended in an ice-cold Tris-magnesium (TM) buffer solution (adjusted to pH 7.4, containing 0.01 M Tris and 0.001 M MgCl_2_) to achieve a concentration of 2.5 × 10^7^ cells/mL. To disrupt the cellular structure, the resuspended cells were mixed with a sucrose solution (1 mol/L) equal to one-third of the cell suspension's volume and sonicated for a duration of 5 min. Subsequently, the homogenized mixture was centrifuged at 700 g for 10 min at 4°C, and the supernatant was separated. This supernatant was then centrifuged again at 14,000 g for 30 min at 4°C, yielding precipitates that contained the collected cell membranes.

### 2.5 Preparation and characterization of GEM@PLGA@HM

For the purpose of membrane coating, the GEM@PLGA particles were blended with macrophage membranes and allowed to incubate at a temperature of 4°C overnight. Subsequently, the mixture underwent sonication for 30 min in an ice bath, followed by extrusion through a 400 nm polycarbonate membrane 20 times. Centrifugation at a speed of 12,000 r/min for 5 min, with water washes in between, was employed to obtain the membrane-coated nanoparticles (NPs). In this context, PLGA@M refers to PLGA NPs coated with unmodified macrophage membranes, whereas PLGA@HM denotes PLGA NPs coated with engineered macrophage membranes that have been modified with anti-HER2 antibodies. The characteristics of the synthesized GEM@PLGA@HM particles, including their average size, potential, surface morphology, and dispersion stability, were evaluated using dynamic light scattering (DLS) and transmission electron microscopy (TEM). The loading of GEM was confirmed by measuring the characteristic UV absorption peak at 269 nm. The macrophage membrane coating was analyzed using SDS-PAGE and TEM.

### 2.6 Detection of *invitro* GEM release

A solution of GEM-loaded PLGA NPs was dialyzed against 50 mL of PBS (either pH 7.4 or pH 5.5) at 37°C using a dialysis membrane with a molecular weight cut-off (MWCO) of 3 kDa. Samples were collected at various time points, including 0, 0.5, 1, 2, 4, 8, 12, 24, and 48 h, with 2 mL of release medium taken each time to measure the amount of GEM present using UV–vis spectroscopy at a wavelength of 269 nm. The calculation formula of GEM release is as follows: GEM release (%) = released GEM quantity/PLGA packed GEM quantity × 100%.

### 2.7 Targeting performance verification *invitro*

HER2-overexpressing cell lines (4T1 and SK-OV-3) and J774A.1 macrophages were plated in six-well plates at a density of 3.5 × 10^5^/mL. Once the cells reached 70% confluence, they were treated with fresh medium containing Rh6G@PLGA, Rh6G@PLGA@M, or Rh6G@PLGA@HM (Rh6G: 0.13 μg/mL). The 4T1 and SK-OV-3 cells were incubated for 1.5 h, while J774A.1 cells were incubated for 0.5 h. After incubation, the cells were fixed and examined under an inverted fluorescence microscope (DMi8, Leica).

For flow cytometry analysis, 4T1, SK-OV-3, and J774A.1 cells were plated in six-well plates (3.5 × 10^5^ cells/mL) and cultured for 24 h. They were then co-incubated with the aforementioned Rh6G-labeled NPs at a concentration of 0.13 μg/mL. The 4T1 and SK-OV-3 cells were incubated for 4 h, while J774A.1 cells were incubated for 1.5 h. Following incubation, the cells underwent centrifugation at 1000 rpm at 4°C for 3 min, washed and resuspended in 200 μL of PBS. The flow cytometry data were collected using a FACS Calibur flow cytometer (BD Biosciences) and subsequently analyzed with FlowJo X10 software (FlowJo).

### 2.8 Anti-tumor efficacy test *invitro*

To assess the safety of GEM@PLGA nanoparticles with J774A.1 cells, a CCK-8 assay was employed to measure cell viability. Once the cell density reached 70%, the medium was switched to a fresh one containing dissociative GEM and GEM@PLGA NPs at concentrations of 0, 0.01, 0.1, 1, 10, and 100 μM/L. The cells were then incubated for durations of 0, 24, 48, and 72 h, followed by conducting the CCK-8 assay.

For evaluating the cytotoxicity against cancer cells, HER2^+^ 4T1 and SK-OV-3 cells were plated in 96-well plates. The medium was replaced with a fresh one containing PBS, GEM@PLGA, PLGA@M, GEM@PLGA@M, PLGA@HM, and GEM@PLGA@HM (GEM: 1 μM/L) when the cell density attained 70%. Subsequently the cells were incubated for 0, 24, 48, and 72 h, and the CCK-8 assay was used to determine the cytotoxicity of GEM-loaded nanoparticles in HER2^+^ tumor cells.

Cells in each treatment group were stained to distinguish between live and dead cells using the Calcein-AM/PI Double Stain Kit. This kit labels live cells with a green fluorescence and dead cells with a red fluorescence. Initially, HER2^+^ 4T1 and SK-OV-3 cells were plated into six-well plates at a density of 3.5 × 10^5^/mL. Once the cells achieved 70% confluence, they were subjected to various treatments including GEM@PLGA, GEM@PLGA@M, GEM@PLGA@HM (each with GEM at 1 μM/L), PLGA@M, PLGA@HM, and a control group treated with PBS, all according to the provided operating manual. Following the treatments, the cells from each group were trypsinized, stained in the dark at 37°C for 15 min, and then examined under a fluorescence microscope.

### 2.9 RT-qPCR and Western blot analysis for the identification of key genes expression

Real-time reverse transcription quantitative PCR (RT-qPCR) experiment was employed to conduct a thorough analysis of the expression profiles of *p53, CCND1, Caspase-3, Ki67, MAPK, AKT, PI3K* (a downstream gene of HER2) in HER2^+^ 4T1 and SK-OV-3 cells following diverse treatments. The specific primers utilized in this work are enumerated in Tables S2 and S3. Cells cultivated in six-well plates were assigned to six distinct treatment cohorts: PBS, GEM@PLGA, PLGA@M, GEM@PLGA@M, PLGA@HM, and GEM@PLGA@HM (GEM: 1 μM/L). Each cohort underwent the designated treatment regimen for 24 h. At the predetermined time point, total RNA was isolated from the cellular lysates using TRIzol reagent, adhering strictly to the manufacturer's protocols. For the purpose of reverse transcription, 1 μg of RNA per sample was meticulously reverse transcribed into cDNA utilizing the PrimeScript RT reagent kit sourced from Takara (Japan). Subsequently, 2 μL aliquots of the synthesized cDNA were subjected to quantitative PCR analysis, targeting the aforementioned genes, using the ABI 7300 Real-Time PCR System (Applied Biosystems, USA). This analysis was conducted in accordance with a previously established and reported methodology [14]. In addition, Western blotting was performed to evaluate the expression of key proteins as previously described [15].

### 2.10 Anti-tumor migration assay *invitro*

The anti-metastasis effect of GEM@PLGA@HM was evaluated by transwell cells migration assay *in* *vitro*. HER2^+^ 4T1-Luc cells were seeded into the upper chamber of transwell plate. When the cells confluence reached 90%, GEM@PLGA@HM and other control groups (PBS, PLGA@M, GEM@PLGA, GEM@PLGA@M and PLGA@HM) were interacted with the cells, the nested upper layer was flipped, and the photos were taken under the microscope after crystal violet staining of the treated cells.

### 2.11 Subcutaneous tumor model establishment

Female BALB/c mice (aged 4-5 weeks, weighing13-15 g) were sourced from Chongqing Tengxin Biological Technology Co., Ltd. These mice were handled in compliance with the guidelines for the care and use of laboratory animals. To induce a HER2-positive 4T1 subcutaneous tumor model, each mouse received a subcutaneous injection of 1 × 10^6^ HER2 positive 4T1 cells into their right hind limb. At the conclusion of the study, the mice were euthanized, and the tumors were excised and weighed.

### 2.12 Targeting performance verification *invivo* and *exvivo*

Once the tumor volume reached 50 mm^3^, the mice were categorized into two distinct groups and administered intravenous injections of either IR780@PLGA@M or IR780@PLGA@HM solutions (both containing 100 μg/mL of IR780). The IVIS imaging system (PerkinElmer IVIS Lumina III) was employed to capture whole-body NIR fluorescence images at different time intervals following the injections (0, 2, 4, 6, 8, 10, 12, 24, and 48 h). To assess the distribution of the NPs within the tissues, the mice were euthanized 48 h post-injection, and their organs and tissues, namely the heart, liver, spleen, lungs, kidneys, and tumor, were harvested for *ex* *vivo* imaging analysis. The fluorescence intensity of the images was then quantified using IVIS software.

### 2.13 Anti-tumor efficacy test *invivo*

Six groups (n = 6), PBS (control group), GEM@PLGA, PLGA@M, GEM@PLGA@M, PLGA@HM and GEM@PLGA@HM, were assembled for tumor therapy. Following anesthesia, mice bearing tumors that had grown to a volume of approximately 50 mm^3^ were administered intravenous injections of either PBS or the aforementioned PLGA nanocarriers (GEM: 2.5 mg/kg). The therapeutic regimen involved three administrations over the study period, with an interval of 2 days between each injection. Tumor volumes and mouse weight were monitored every 2 days post-treatment. The mice were sacrificed by the end of the study, and their tumor tissues were photographed and weighed. To further elucidate the *in* *vivo* anti-tumor mechanisms of GEM@PLGA@HM, immunohistochemistry (IHC) was carried out following the standard protocol described in the literature [16]. Meanwhile, RNA was extracted from the *ex* *vivo* tumor tissues, and RT-qPCR analysis was performed to assess the expression levels of key genes, including *p53, CCND1, Caspase-3, Ki67, MAPK, AKT,* and *PI3K*, in the tumor tissues from each treatment group. All animal experiments conducted in this study adhered to the protocols approved by the Laboratory Animal Welfare and Ethics Committee of Air Force Medical University (approval number: 20200316).

The *in* *vivo* safety of all drugs was assessed through hematological and histological analyses. Blood samples were collected from mice undergoing tumor therapy, centrifuged, and the resulting serum was utilized to measure the levels of alanine aminotransferase (ALT), aspartate aminotransferase (AST), albumin (ALB), blood urea nitrogen (BUN), creatinine (CR), and uric acid (UA). Histological examination was conducted using Hematoxylin and Eosin (H&E) staining, following standard experimental protocols described in previous report [17].

### 2.14 Anti-tumor metastasis assay *invivo*

A HER2^+^ 4T1-Luc tumor model of breast cancer was constructed to evaluate the anti-metastasis effect of GEM@PLGA@HM in mice. 5 × 10^5^ HER2 positive 4T1-Luc cells were intravenously injected into the mice. The 30 mice were divided into six groups (n = 5), PBS (control group), GEM@PLGA, PLGA@M, GEM@PLGA@M, PLGA@HM and GEM@PLGA@HM. On the 5, 10 and 15th day after injection, each tumor-bearing mouse was intravenously injected with 100 μL PBS or different NPs (GEM: 2.5 mg/kg). Luciferase substrate dissolved in DPBS with a concentration of 15 mg/mL was prepared. The amount of substrate that each mouse should receive was calculated according to the standard of 10 μL/g, and then intraperitoneally injected into the mouse. IVIS software was used to monitor the bioluminescence of HER2^+^ 4T1-Luc tumor and to track the growth and development of tumor metastasis *in vivo*.

### 2.15 *Exvivo* imaging of lung metastatic tumor lesions

When finishing the metastatic tumors therapy, the mice were sacrificed and dissected. Subsequently, the mice organs/tissues, including the heart, liver, spleen, lung, kidney were collected for *ex* *vivo* imaging. In particular, lung tissue was placed separately on filter paper and then photographed. The metastatic tumor lesions on the lung surface of mice in each treatment group and control group were carefully examined and counted. The lung tissue was subsequently used to make sections for H&E staining and imaging.

### 2.16 Monitoring of body weight and survival curve of mice

During the therapy, mice in all treatment groups and control group were weighed once every 5 days. Weight data and survival rates of mice were recorded, from which line plots depicting weight changes and survival curves were subsequently generated. Notably, variations in the body weight of the mice served as an indicator to ascertain whether metastatic tumors had progressed to a stage that could potentially compromise their health.

### 2.17 Statistical analysis

Data from at least three independent experiments were subjected to statistical analysis. The experimental results were presented in the form of mean ± SD. For pairwise comparisons between groups, a two-tailed Student's t-test was employed. Multiple comparisons were analyzed using ANOVA. Statistical significance was established at a *p* value of less than 0.05.

## 3 Results

### 3.1 Synthesis and characterization of HER2 antibody modified macrophage membrane-camouflaged NPs

**Fig. 1:**
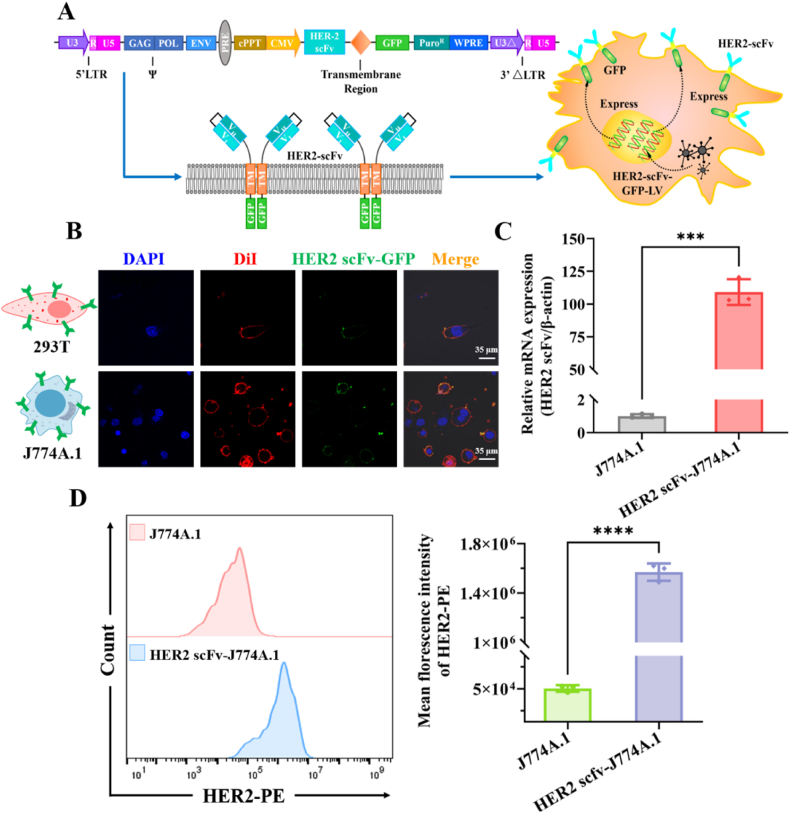
Construction and characterization of HER2 antibody-armed macrophage membrane. (A) Schematic illustration of the preparation process of engineered macrophage expressing HER2 antibody. Lentivirus (LV) system was constructed by which an anti-HER2 antibody was genetically engineered to sprout from the macrophage membrane, and the transmembrane (TM) domain and GFP were designed to postfix the anti-HER2 antibody with linker so that it was armed on macrophages. (B) The confocal imaging of macrophages expressing HER2 scFv-GFP. The results showed that the green fluorescence (HER2 scFv-GFP) overlapped with the red fluorescence (DiI dye), which demonstrated the transmembrane localization of HER2 scFv-GFP. Scale bar = 35 μm. (C) The expression of HER2 scFv-GFP was verified by qRT-PCR analysis at the level of transcription. (D) Assessment of HER2-binding affinity and quantitative results of transmembranously expressed HER2-scFv by flow cytometry. ∗∗∗ and ∗∗∗∗ represent *p* < 0.001 and *p* < 0.0001, respectively (For interpretation of the references to colour in this figure legend, the reader is referred to the Web version of this article).

Previous studies have shown that HER2 is significantly overexpressed in various cancers, especially in breast cancer (BRCA) and stomach adenocarcinoma (STAD). Consequently, cancers exhibiting high HER2 expression were chosen as the target cancer models for our nanodrug delivery system. A lentiviral vector system was engineered to express an anti-HER2 antibody (HER2-scFv) by genetically modifying it to emerge from the membrane. Additionally, transmembrane (TM) sequences were incorporated to link the HER2-scFv, enabling its attachment to macrophages (Fig. 1A). A green fluorescent protein (GFP) was fused with the TM and HER2-scFv sequences to be expressed on the inner surface of the cell membrane. Following lentiviral transfection, the cell membrane was stained with the dye DiI and visualized using confocal scanning microscopy. As anticipated, the results showed abundant HER2-scFv linked to GFP (green fluorescence) on the cell membranes (labeled with red fluorescence) of both HEK 293T and J774A.1 macrophages after lentiviral transfection (Fig. 1B). Quantitative polymerase chain reaction (qPCR) analysis demonstrated the overexpression of HER2-scFv (Fig. 1C). Flow cytometry results (Fig. 1D) indicated that J774A.1 cells, which had been stably transfected with the HER2-scFv gene, displayed a 31-fold increase in red fluorescence intensity compared to unmodified J774A.1 cells following incubation with PE-labeled HER2 protein, further substantiating the stable membrane expression of HER2-scFv and its preserved functional HER2-binding affinity. These findings indicate the stable expression and fusion of the HER2-scFv-GFP plasmid on the exterior of macrophages, confirming the successful construction of anti-HER2 macrophages (HM).

**Fig. 2:**
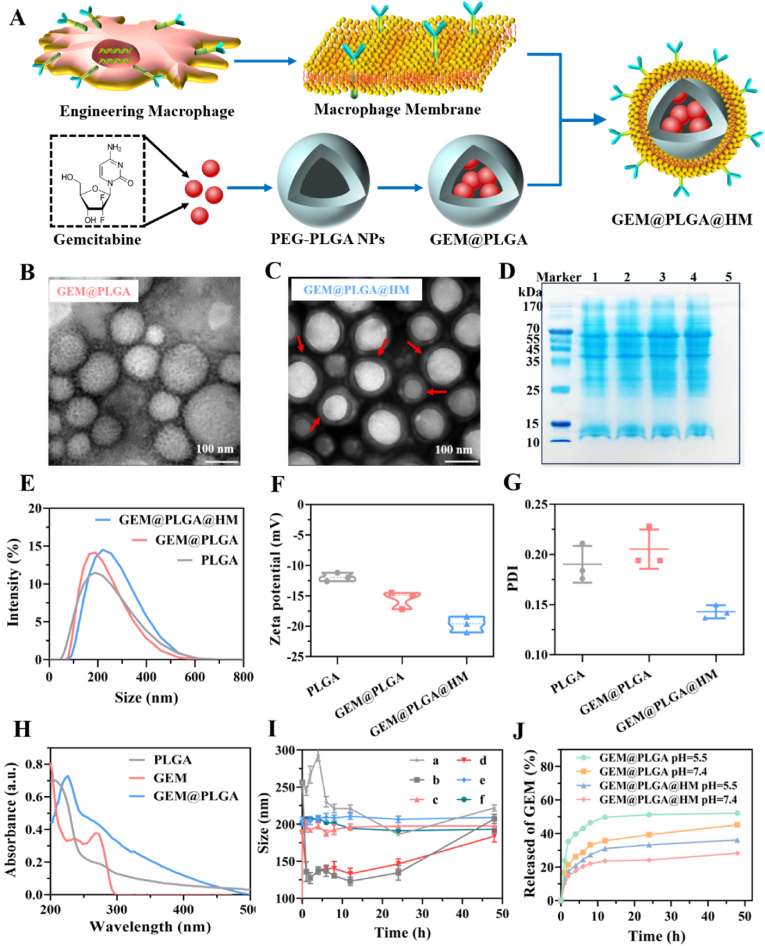
Construction and characterization of PLGA NPs coated with HER2 antibody-armed macrophage membrane. (A) Schematic illustration of the preparation process of GEM@PLGA@HM. (B, C) Representative TEM image of GEM@PLGA and GEM@PLGA@HM. Scale bar = 100 nm. (D) The normal and engineered macrophage membrane coatings were analyzed using SDS-PAGE. (E) PLGA core, GEM@PLGA and GEM@PLGA@HM were showed their size distribution by DLS. (F, G) The surface zeta potential and PDI of PLGA core, GEM@PLGA and GEM@PLGA@HM were confirmed (n = 3, mean ± SD). (H) Fluorescence spectra of the PLGA core, GEM, and GEM@PLGA. (I) The sizes of PLGA core, GEM@PLGA and GEM@PLGA@HM in PBS or FBS at different times, (a) NPs in PBS, (b) NPs in FBS, (c) GEM@PLGA in PBS, (d) GEM@PLGA in FBS, (e) GEM@PLGA@HM in PBS, (f) GEM@PLGA@HM in FBS. (J) *Invitro* release profiles of GEM from GEM@PLGA (pH = 5.5, 7.4) and GEM@PLGA@HM NPs (pH = 5.5, 7.4) at different times.

In order to improve the stability and biosafety of drug delivery, the optimal synthesis scheme with ultrasound 1 intensity of 75 W and ultrasound 2 intensity of 100 W was adopted to synthesize GEM@PLGA (Table S1). In this ultrasonic mode, the synthesized nanoparticles with the smallest size and PDI value were stable and suitable for drug delivery (Table S1 and Fig. S1). The standard curve of GEM at 269 nm UV absorption was measured and drawn to calculate the encapsulation rate of nanoparticles (Fig. S2). The entire preparation process of GEM@PLGA@HM is presented in the form of a schematic illustration in Fig. 2A. Transmission electron microscopy (TEM) images provided clear evidence of a membrane layer enveloping the PLGA core (Fig. 2C), contributing to a smoother NP surface and a more homogeneous particle size distribution (Fig. 2B and C, and S3). In addition, through the polyacrylamide gel electrophoresis experiment, it can be obviously observed that the protein bands in the four groups of samples (normal macrophage membrane, GEM@PLGA@M, engineered macrophage membrane and GEM@PLGA@HM) were located at the similar strip positions, except for GEM@PLGA, indicating that the cell membrane proteins of engineered macrophages were perfectly preserved on the PLGA nanoparticles (Fig. 2D). When successfully synthesized, PLGA NPs with or without GEM loading, demonstrated a consistent size distribution centered at approximately 190 nm, as verified through dynamic light scattering (DLS) analysis (Fig. 2E). Upon coating with HER2-scFv-armed macrophage membranes, the resultant GEM@PLGA@HM NPs exhibited a marginally larger average diameter compared to their uncoated PLGA core counterparts or GEM@PLGA NPs (Fig. 2E). Since GEM and HER2-scFv-armed macrophage membranes were gradually loaded onto the PLGA cores, the GEM@PLGA@HM displayed a surface charge of roughly −19.7 mV, which was more negative than that of GEM@PLGA@M (−15.5 mV) and the PLGA cores alone (−11.9 mV) (Fig. 2F). Compared with PLGA core or GEM@PLGA, the polydispersity index (PDI) values of GEM@PLGA@HM copolymers decreased to 0.143, aligning closely with the theoretical value for normal polydispersity, indicating a favorable degree of monodispersity for these engineered macrophage membrane-coated NPs (Fig. 2G). Collectively, these findings indicate the successful and preserved coating of engineered macrophage membranes in this study.

The ultraviolet–visible (UV) absorption spectra revealed that both GEM and GEM@PLGA exhibited absorption peaks near 269 nm (Fig. 2H), whereas no distinct absorption peak was noted for the unladen PLGA NPs. These observations confirmed the successful encapsulation of GEM within the PLGA core. To assess the stability of the NPs, GEM@PLGA and GEM@PLGA@HM were suspended in fetal bovine serum (FBS) and phosphate-buffered saline (PBS). Within 48 h, GEM@PLGA@HM demonstrated superior stability in both media, with particle size fluctuations generally hovering around 210 nm (The e and f line charts in Fig. 2I). In contrast, the uncoated NPs and GEM@PLGA samples showed significant size variations when exposed to FBS (The b and d line charts in Fig. 2I), emphasizing the crucial role of macrophage membrane coating in enhancing nanocarrier stability. To evaluate drug release profiles, GEM@PLGA and GEM@PLGA@HM were dialyzed at various pH levels, and the drug retention within the NPs was quantified over time. The results indicated that both GEM@PLGA and GEM@PLGA@HM continuously released GEM under different pH conditions (Fig. 2J). Notably, drug release was higher in an acidic environment (pH 5.5) compared to neutral pH (Fig. 2J). Under identical conditions, the membrane-coated NPs exhibited lower drug release rates than the uncoated ones (Fig. 2J). These findings suggest that the macrophage membrane encapsulation not only enhances NP stability but also acts as a diffusion barrier for GEM release, facilitating controlled and accelerated drug delivery into the acidic tumor microenvironment.

### 3.2 HER2^+^ tumor targeting efficiency of GEM@PLGA@HM *invitro*

Standard curves for the rhodamine 6G (Rh6G) fluorescent dye were established to facilitate the subsequent standardization and analysis of experimental procedures (Fig. S4). UV–visible absorption spectra confirmed the successful encapsulation of Rh6G within the PEG-PLGA core, as both Rh6G and Rh6G@PEG-PLGA exhibited absorption peaks at 530 nm, while PEG-PLGA NPs alone showed no specific absorption peak (Fig. S5).

**Fig. 3:**
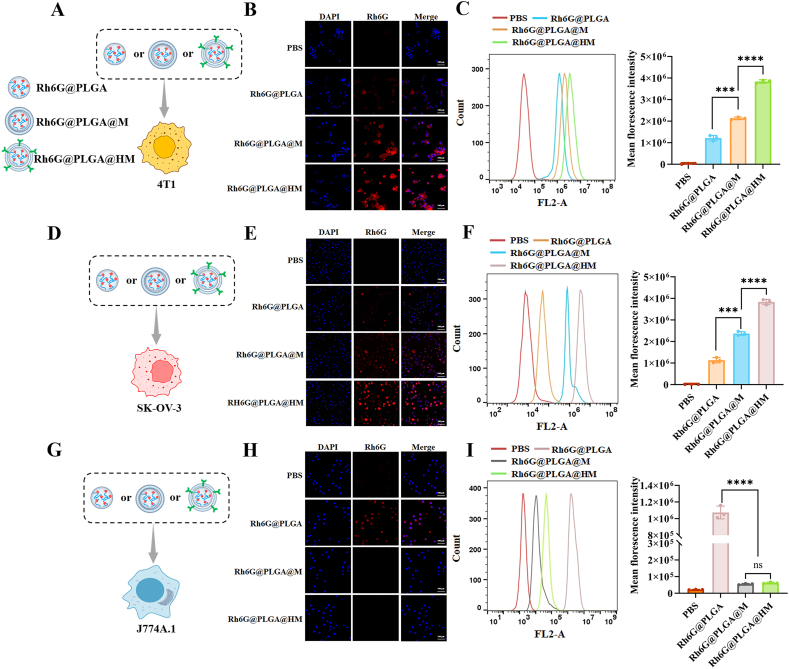
Cellular uptake and biomimetic characterization of Rh6G@PLGA@HM. Schematic diagram of (A, D) two types of cancer cells, and (G) one kind of normal cells treated with Rh6G@PLGA, Rh6G@PLGA@M, and Rh6G@PLGA@HM separately. Confocal microscopic images of (B) HER2 positive 4T1 cells, (E) HER2 positive SK-OV-3 cells were incubated for 1.5 h, and (H) J774A.1 cells were incubated for 0.5 h with Rh6G@PLGA, Rh6G@PLGA@M, and Rh6G@PLGA@HM. Scale bar = 100 μm. (C, F and I) Flow cytometry analysis and quantitative results of (C) 4T1 cells, (F) SK-OV-3 cells and (I) J774A.1 cells incubated with the above nanoparticles.

To assess the HER2^+^ tumor targeting efficiency at the cellular level, cancer cells (4T1 and SK-OV-3 overexpressing HER2) and normal cells (J774A.1 macrophages) were utilized, as illustrated in Fig. 3A, D, and 3G. A gradual increase in fluorescence intensity was observed from the control (PBS), Rh6G@PLGA, Rh6G@PLGA@M, and Rh6G@PLGA@HM in both 4T1 (Fig. 3B) and SK-OV-3 cells (Fig. 3E). Notably, Rh6G@PLGA@M exhibited significantly enhanced intracellular fluorescence compared to Rh6G@PLGA, indicating the natural affinity of macrophage membranes to cancer cells and facilitating the effective uptake of Rh6G@PLGA@M by these cells. The highest Rh6G uptake was achieved with Rh6G@PLGA@HM, suggesting that HER2-scFv-armed macrophage membranes significantly improve the targeting of NPs towards HER2^+^ tumor cells and enhance their phagocytosis by tumor cells. In contrast, when incubated with J774A.1 macrophages, Rh6G@PLGA@M and Rh6G@PLGA@HM showed reduced fluorescence compared to Rh6G@PLGA (Fig. 3H), indicating that the biomimetic properties of macrophage membranes help NPs evade capture by immune cells. These findings align with previous reports that ICG/DOX@PLGA NPs, wrapped with anti-HER2 affibody-armed macrophage membranes, showed excellent HER2-targeting capabilities and enhanced the probability of contact between NPs and tumor cells [18]. The flow cytometry results aligned with the aforementioned findings, revealing a sequential increase in fluorescence intensity across 4T1 and SK-OV-3 cells treated with PBS, Rh6G@PLGA, Rh6G@PLGA@M, and Rh6G@PLGA@HM (Fig. 3C and F). Among these, the Rh6G@PLGA@HM group demonstrated the highest fluorescence intensity in both tumor cell lines, being 1.80–3.16-fold (4T1 cells) and 1.62–3.39-fold (SK-OV-3 cells) greater than the Rh6G@PLGA@M and Rh6G@PLGA groups, respectively. Conversely, macrophage J774A.1 cells exhibited significantly reduced fluorescence when treated with Rh6G@PLGA@M and Rh6G@PLGA@HM, falling below the levels observed in the Rh6G@PLGA group and approaching those seen the PBS group (Fig. 3I). These outcomes corroborate the data obtained from confocal imaging, confirming that HER2-scFv-modified macrophage membrane camouflage NPs acquire specific tumor-targeting and biomimetic properties, which are critical for efficient drug delivery to HER2^+^ tumor cells.

### 3.3 Anti-tumor effects of GEM@PLGA@HM *invitro*

**Fig. 4:**
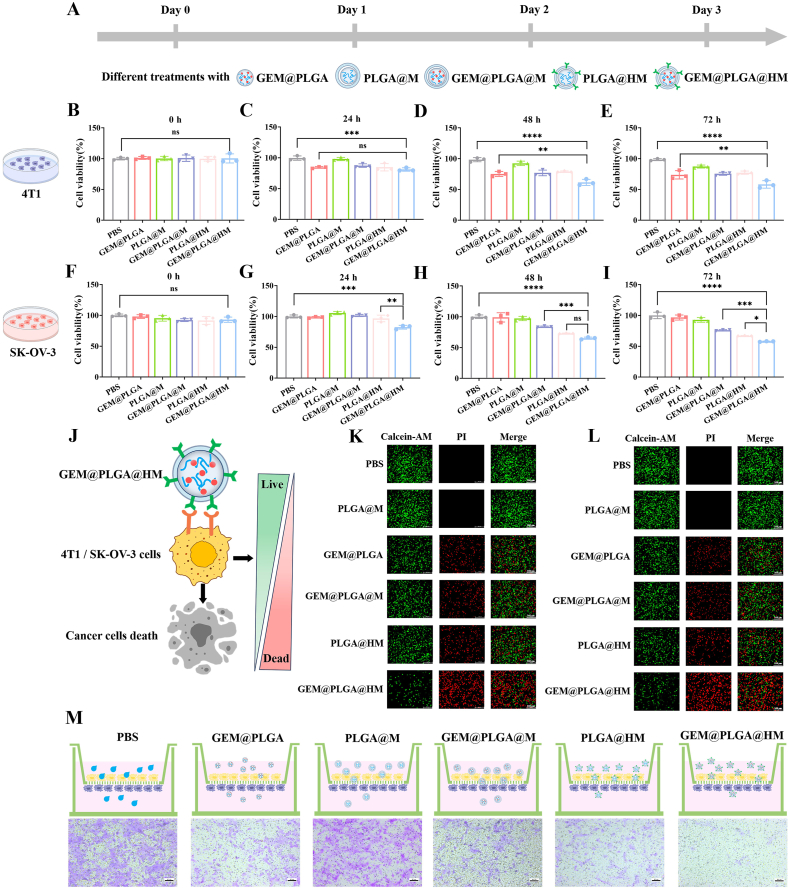
*Invitro* cytotoxicity and analysis of anti-metastasis effect. (A) Schematic diagram of cells treatment. Cell viability analysis of (B–E) HER2^+^ 4T1 and (F–I) SK-OV-3 after incubation with PBS, GEM@PLGA, PLGA@M, GEM@PLGA@M, PLGA@HM and GEM@PLGA@HM (GEM: 1 μM/L) for 0, 24, 48 and 72 h separately. (J) Schematic diagram of live/dead cells identification by Calcein-AM/propidium iodide (PI) double staining. The confocal microscopic images of (K) HER2^+^ 4T1 and (L) SK-OV-3 cells were treated with PBS, GEM@PLGA, PLGA@M, GEM@PLGA@M, PLGA@HM and GEM@PLGA@HM (GEM: 1 μM/L) separately. Data are expressed as mean ± SD (n = 3), and all statistics are analyzed by one-way ANOVA. Scale bar = 166 μm. (M) Transwell cells migration assay. HER2^+^ 4T1-Luc cells were interacted with PBS, PLGA@M, GEM@PLGA, GEM@PLGA@M, PLGA@HM and GEM@PLGA@HM. The photos were taken under the optical microscope after crystal violet staining of the treated cells. Scale bar = 50 μm. The data are presented as means ± SD. ∗, ∗∗, ∗∗∗ and ∗∗∗∗ represent *p* < 0.05, *p* < 0.01, *p* < 0.001 and *p* < 0.0001, respectively. (For interpretation of the references to colour in this figure legend, the reader is referred to the Web version of this article.)

After confirming the tumor targeting and biomimetic properties of PLGA nanoparticles (NPs) coated with macrophage membranes armed with HER2-scFv, gemcitabine (GEM) was chosen as the nanocarrier's core. For safety assessments, J774A.1 cells were utilized, while 4T1 and SK-OV-3 cells were selected to evaluate the toxicity of various NPs against cancer cells over time (Fig. 4A). In terms of safety, both free GEM and GEM@PLGA were incubated with J774A.1 cells for durations of 0, 24, 48, and 72 h, across various GEM concentrations (Figs. S6 and S7). Notably, after 24 h of incubation, the PLGA NP-encapsulated GEM exhibited no significant cytotoxicity at concentrations of 1 μM or below (Fig. S7). Conversely, free GEM induced cell death at comparable doses (Fig. S6). Consequently, a GEM concentration of 1 μM was selected for subsequent experiments, ensuring that GEM@PLGA did not elicit adverse reactions during drug delivery but released adequate GEM to exert an anti-tumor effect upon reaching the tumor site. Cytotoxicity tests revealed that GEM@PLGA@HM (GEM at 1 μM/L) exhibited significantly higher cytotoxicity in HER2^+^ 4T1 cells (Fig. 4B–E) and SK-OV-3 cells (Fig. 4F–I) compared to other groups, in a time-dependent manner (Figs. S8 and S9). This indicated superior tumor cell targeting by GEM@PLGA@HM. Furthermore, both GEM@PLGA@M and PLGA@HM displayed moderate cytotoxic effects in both cell lines.

To visually assess the cytotoxic efficacy of various treatments, including PBS, PLGA@M, GEM@PLGA, GEM@PLGA@M, PLGA@HM, and GEM@PLGA@HM (GEM:1 μM/L), a live/dead double staining assay was employed (Fig. 4J). Live 4T1 (Fig. 4K) and SK-OV-3 cells (Fig. 4L) were stained green with calcein acetoxymethyl ester (calcein-AM), while dead cells were stained red with propidium iodide (PI). As anticipated, GEM@PLGA@HM demonstrated the highest cytotoxicity among all groups, with a significant increase in the red fluorescent signal indicating dead 4T1 and SK-OV-3 cells compared to GEM@PLGA, GEM@PLGA@M, and PLGA@HM. These findings aligned with the results of the CCK8 cytotoxicity assay, confirming that GEM@PLGA@HM effectively induced the death of HER2^+^ tumor cells. Furthermore, the migration ability of HER2^+^ tumor cells was evaluated, with the results presented in Fig. 4M. Red fluorescence indicated the successful migration of 4T1-Luc tumor cells through the membrane to the bottom of the transwell's upper chamber. Notably, the GEM@PLGA@HM-treated group exhibited the weakest red fluorescence, suggesting its superior anti-metastatic effect on HER2^+^ 4T1 tumor cells compared to other groups.

### 3.4 *Invitro* anti-tumor mechanisms of GEM@PLGA@HM

**Fig. 5:**
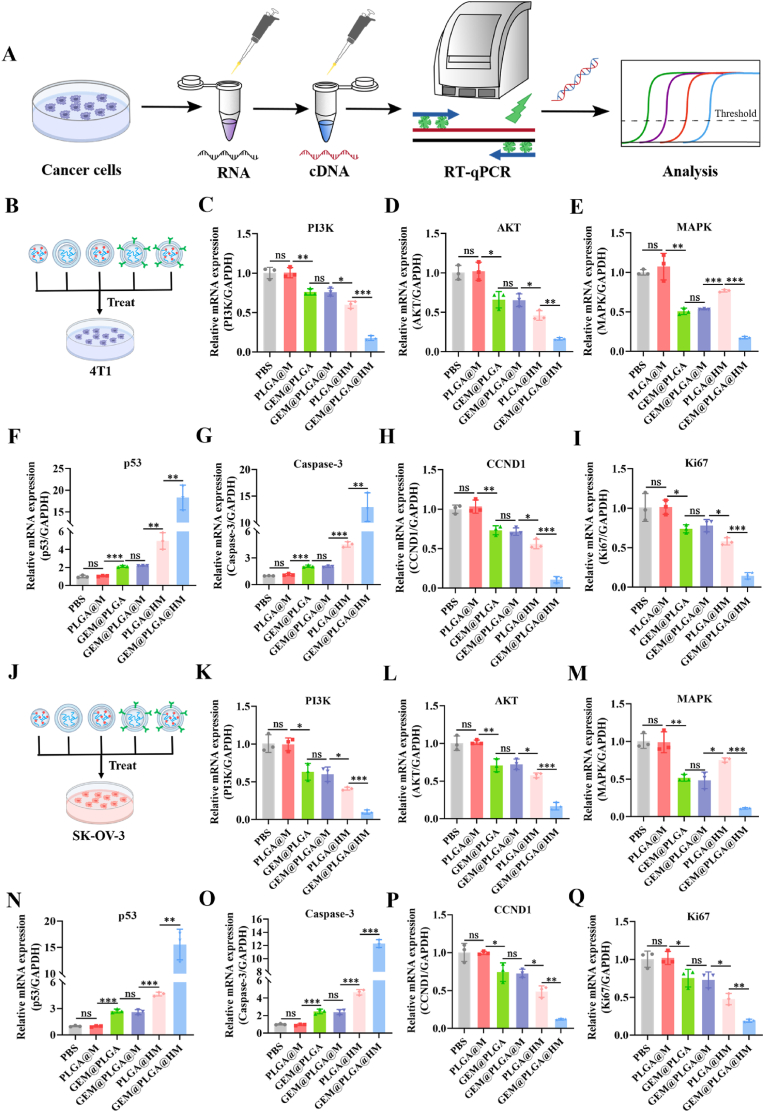
Changes in the expression of key genes in HER2^+^ 4T1 cells and SK-OV-3 cells after treatments. (A) Schematic diagram of genes purification and assessment in treated cancer cells by RT-qPCR. (B–I) The expression of (C) *PI3K*, (D) *AKT*, (E) *MAPK*, (F) *Pp53*, (G) *Caspase-3*, (H) *CCND1*, and (I) *Ki67* in (B) 4T1 cells treated with PBS, PLGA@M, GEM@PLGA, GEM@PLGA@M, PLGA@HM and GEM@PLGA@HM (GEM: 1 μM/L) for 24 h, respectively. (J–Q) The expression of (K) *PI3K*, (L) *AKT*, (M) *MAPK*, (N)*p53*, (O) *Caspase-3*, (P) *CCND1*, and (Q) *Ki67* in (J) SK-OV-3 cells treated with PBS, PLGA@M, GEM@PLGA, GEM@PLGA@M, PLGA@HM and GEM@PLGA@HM (GEM: 1 μM/L) for 24 h, respectively. The data are presented as means ± SD. ∗, ∗∗, and ∗∗∗ represent *p* < 0.05, *p* < 0.01, and *p* < 0.001, respectively.

Given the augmented tumoricidal efficacy demonstrated by GEM@PLGA@HM *in* *vitro*, we delved deeper into the underlying mechanisms responsible for its cancer-targeting and inhibitory capabilities, employing HER2^+^ 4T1 and SK-OV-3 cells as our model systems (Fig. 5A). GEM (2′, 2′-difluorodeoxycytidine, dFdC) is a nucleoside analogue of deoxycytidine, which can be phosphorylated and activated by intracellular deoxycytidine kinase, forming the active metabolites gemcitabine diphosphate (dFdCDP) and gemcitabine triphosphate (dFdCTP). dFdCDP inhibits ribonucleotide reductase that is responsible for producing the deoxynucleotides required for DNA synthesis and repair. dFdCTP competes with cellular deoxynucleotides (particularly dCTP) for incorporation into DNA, thereby inhibiting DNA replication and inducing DNA damage. Through these dual functions, GEM can lead to fragmentation and subsequent death of cancer [19]. The unique combination of metabolic properties and mechanistic characteristics of GEM suggests that GEM is likely to be synergistic with other drugs or other pathways that damage DNA. The Phosphoinositide-3 kinase (PI3K)/AKT signaling pathways have been shown to play a pivotal role in regulating cell proliferation and survival by inhibiting apoptosis and advancing the cell cycle [20]. Furthermore, It has been documented that an increase in HER2 gene expression leads to the activation of downstream PI3K/AKT pathways, ultimately accelerating tumor progression [21]. Therefore, changes in the expression of key genes (*PI3K, AKT, MAPK,* *p53, Caspase-3, CCND1,* and *Ki67*) in 4T1 cells or SK-OV-3 cells after PBS, PLGA@M, GEM@PLGA, GEM@PLGA@M, PLGA@HM and GEM@PLGA@HM (GEM: 1 μM/L) treatments were measured by real time quantitative PCR (Fig. 5B−5I, 5J-5Q). The current findings suggest that the combination of HER2-scFv and gemcitabine (GEM), delivered via GEM@PLGA@HM treatment, resulted in a notable suppression of the PI3K (Fig. 5C and K) and AKT genes (Fig. 5D and L) within the PI3K/AKT signaling cascade. Previous studies have highlighted the important role of mitogen of activated protein kinase (MAPK) in cell proliferation through the activation of the Ras-Raf-MAPK pathway and the transcription factors NF-κB and CREB, which are often overexpressed or overactivated in tumors and further regulate the expression of downstream gene like *p53* to promote cell proliferation [22,23]. In the present study, the expression of the *MAPK* gene in cells subjected to GEM@PLGA@HM treatment exhibited a marked reduction (Fig. 5E and M). Additionally, when compared to other groups, the levels of *p*53 gene expression (Fig. 5F and N) were significantly elevated in both cell types following GEM@PLGA@HM treatment. This upregulation of *p*53 could effectively induce cell cycle arrest, decrease cell proliferation, and enhance apoptosis in cancer cells. This was further validated by the increased expression of *Caspase-3* (Fig. 5G and O), while the notable downregulation of *CCND1* (Fig. 5H and P) and *Ki67* (Fig. 5I and Q) expressions. These alterations can be ascribed to the regulation of *p53*, which significantly induces apoptosis, cell cycle arrest, and inhibition of cell proliferation. The expression levels of crucial proteins in relevant signaling pathways, such as MAPK, *p*53, and Caspase-3, were further investigated using Western Blotting (Fig. S10). The results demonstrated that the combined effect of GEM and HER2-scFv synergistically inhibits MAPK expression while enhancing the expression levels of *p53* and *Caspase-3*, which provides additional validation for the RT-qPCR results mentioned above.

Consequently, GEM@PLGA@HM demonstrated potent anti-tumor effects against HER2^+^ tumors, potentially due to the synergistic impact of increased intracellular GEM delivery and the inhibition of the HER2-induced signaling pathway by the HER2-scFv. These findings provide a theoretical foundation for the subsequent treatment of HER2^+^ tumor models in mice.

### 3.5 HER2^+^ tumor targeting performance of IR780@PLGA@HM *invivo*

**Fig. 6:**
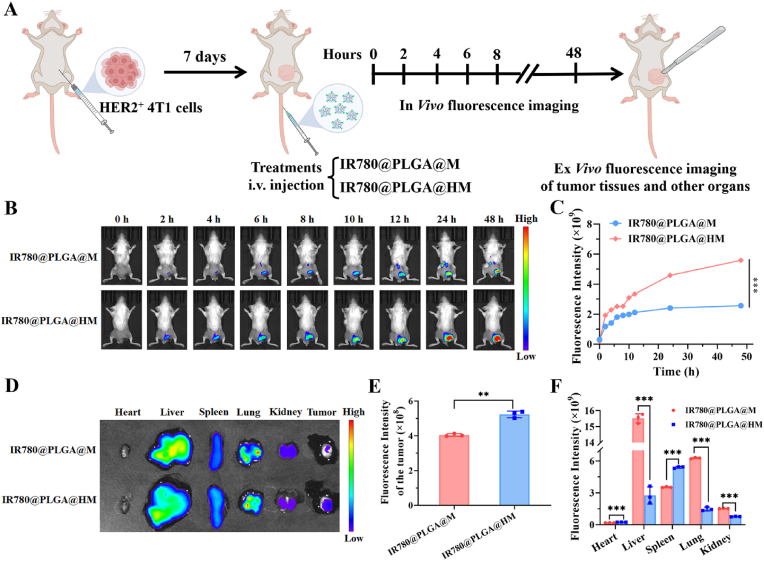
*Invivo* tumor-targeted fluorescence imaging and biodistribution of HER2 antibody-modified membrane camouflaging PLGA NPs. (A) Schematic illustration of *invivo* and *exvivo* imaging of PLGA NPs delivery using a subcutaneous breast tumor model and IVIS imaging. (B) Representative NIR fluorescence images of HER2 positive 4T1 tumor bearing mice after tail vein injection with IR780@PLGA@M or IR780@PLGA@HM (IR780: 100 μg/mL) for different times (0, 2, 4, 6, 8, 10, 12, 24 and 48 h). (C) Quantitative analysis of fluorescence signals of the *invivo* tumor regions in IR780@PLGA@M and IR780@PLGA@HM treated mice over injection time as in (B). (D) Fluorescence images of the *exvivo* primary organs (heart, liver, spleen, lungs, and kidneys) and tumors in the IR780@PLGA@M and IR780@PLGA@HM groups 48 h post-injection. (E) Quantitative analysis of fluorescence intensity of the *exvivo* tumor regions and (F) major organs (heart, liver, spleen, lungs, and kidneys) as in (D). The data are presented as means ± SD. ∗, ∗∗, and ∗∗∗ represent *p* < 0.05, *p* < 0.01, and *p* < 0.001, respectively.

IR780 is a widely used near-infrared dye with an excitation wavelength of about 780 nm, which is provided with high penetrability in living organisms, making IR780 ideal for near-infrared imaging *in* *vivo*. To investigate the specific targeting capability of HER2-scFv-modified membrane camouflaged PLGA nanoparticles (NPs) towards HER2^+^ tumors *in* *vivo*, we incorporated the IR780 dye into the core of the PLGA NP carrier, which allowed to visualize the distribution of PLGA@M and PLGA@HM using IVIS imaging systems. IR780-labeled PLGA@M and PLGA@HM were administered intravenously to mice bearing HER2^+^ 4T1 tumors. The *in* *vivo* distribution of IR780@PLGA@M and IR780@PLGA@HM at various time points within 48 h were analyzed (Fig. 6A). Our results revealed a gradual accumulation of IR780 fluorescence in the tumor regions of mice treated with the nanocarriers over time. Notably, the fluorescence intensity in the IR780@PLGA@HM group was significantly higher than that in the IR780@PLGA@M group (Fig. 6B and C). This indicated that the HER2-scFv-engineered macrophage membrane significantly enhanced the targeting of PLGA NPs to HER2^+^ tumors, resulting in more efficient enrichment of the nanocarrier at the tumor site.

Furthermore, we dissected the mice to perform *ex* *vivo* imaging. The results clearly demonstrated that in the IR780@PLGA@HM-treated group, there was a significant increase in fluorescence accumulation in the tumors (Fig. 6D and E). Additionally, the levels of IR780 were reduced in most tissue organs, particularly in the liver, lungs, and kidneys (Fig. 6D and F).

Collectively, the findings from both *in* *vitro* and *in* *vivo* experiments underscored the remarkable tumor targeting capabilities of GEM@PLGA@HM, attributed to the synergistic effects of the engineered HER2 antibody and the biomimetic macrophage membranes. This laid the groundwork for the enrichment of GEM in tumors and further targeted therapeutic applications.

### 3.6 Programmed HER2^+^ tumor targeted therapy of GEM@PLGA@HM *invivo*

**Fig. 7:**
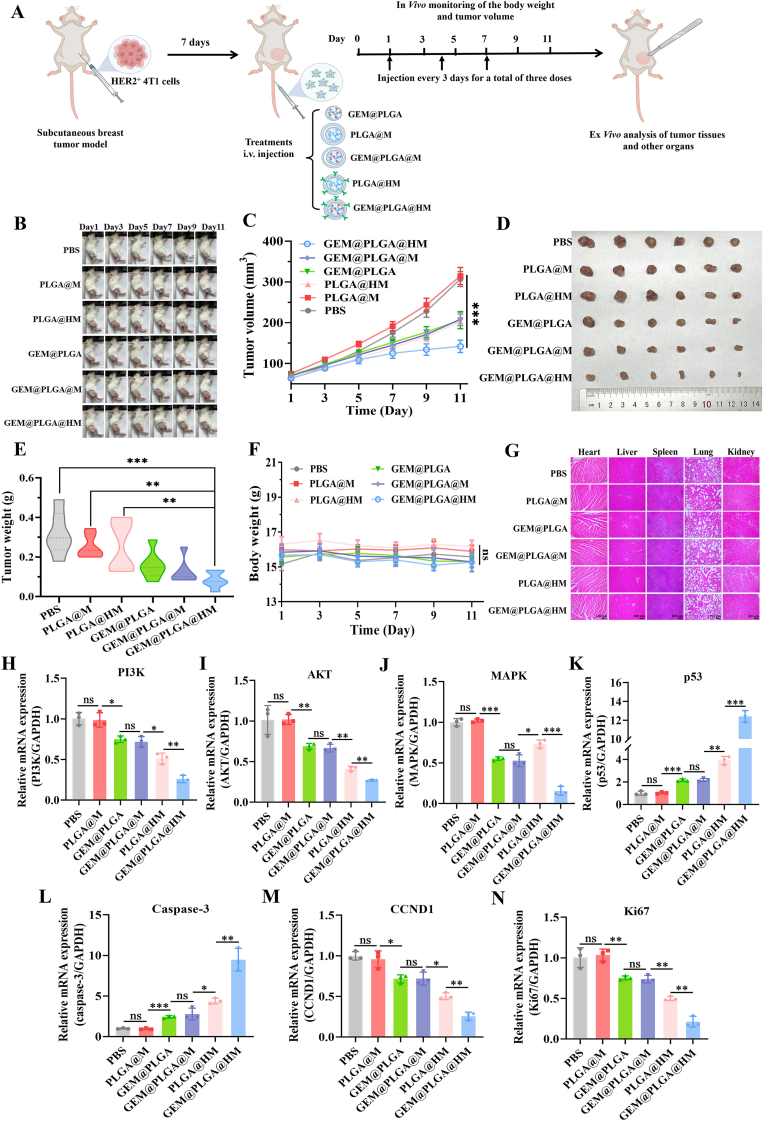
*Invivo* therapeutic effect and mechanism of GEM@PLGA@HM for HER2 positive 4T1 tumor. (A) Schematic illustration of treatment schedule, *invivo* monitoring and *exvivo* analysis using a subcutaneous breast tumor model. (B) Changes of tumor appearance, (C) tumor growth curves, (D) isolated tumor volume and (E) tumor weights of HER2 positive 4T1 bearing mice administrated with PBS, PLGA@M, GEM@PLGA, GEM@PLGA@M, PLGA@HM and GEM@PLGA@HM respectively in the unilateral tumor experiment models (n = 6). *Invivo* safety evaluation of different nano-carriers in unilateral HER2 positive 4T1 models (n = 6) by monitoring (F) the changes of relative body weights and observing (G) H&E staining sections of major organs including heart, liver, spleen, lungs, and kidneys. Scale bar = 100 μm. (H–N) The RT-qPCR analysis of seven classic genes in tumor tissues, (H) *PI3K*, (I) *AKT*, (J) *MAPK*, (K) *p53*, (L) *Caspase-3*, (M) *CCND1*, and (N) *Ki67*. The data are presented as means ± SD. ∗, ∗∗, and ∗∗∗ represent *p* < 0.05, *p* < 0.01, and *p* < 0.001, respectively.

A mouse subcutaneous breast tumor model was established for further evaluating the *in* *vivo* efficacy of HER2-scFv-armed macrophage membrane camouflaged GEM@PLGA@HM and other PLGA NPs (Fig. 7A). The animals were divided in to six groups: (I) control (administered PBS 200 μL); (II) PLGA@M; (III) PLGA@HM; and (IV) GEM@PLGA; (Ⅴ) GEM@PLGA@M; (Ⅵ) GEM@PLGA@HM. These nano-carriers were administered intravenously to the HER2^+^ 4T1 model mice (n = 6 per group).

Following three drug administrations, the appearance and growth of tumors in each group were meticulously monitored. The tumor morphology and growth curves are depicted in Fig. 7B and C. When compared to the control and PLGA@M groups, tumors in the GEM@PLGA, GEM@PLGA@M, and PLGA@HM treatment groups exhibited a slower growth rate. This was attributed to the chemotherapy effect of GEM released from the PLGA NPs or the targeted inhibitory performance of HER2^+^ tumors mediated by the engineered HER2 antibody.

With the prolongation of the therapeutic process, tumors in the PBS and PLGA@M groups grew rapidly compared to those treated with other NPs. Notably, on Day 9, tumor growth slowed down significantly in the GEM@PLGA@HM group, whereas tumors in other treatment groups continued to grow to varying degrees. After 11 days of therapy, tumors in GEM@PLGA@HM-treated mice were markedly inhibited and exhibited the smallest tumor volume compared to other groups (Fig. 7B and C). This was likely due to the prolonged circulation of NPs coated with the macrophage membrane, the specific targeting of HER2 antibodies to HER2^+^ tumors, and accelerated GEM release from NPs enriched in tumors, thereby achieving precise and exceptional combined therapeutic efficacy.

Post-treatment, mice were dissected, and their tumors were excised and photographed (Fig. 7D). Notably, the mice administered with GEM@PLGA@HM exhibited the most reduced tumor volumes among all treated cohorts, underscoring the superior tumor-suppressing efficacy facilitated by GEM@PLGA@HM. To substantiate these findings, the excised tumors were weighed *ex* *vivo* (Fig. 7E), revealing a significantly lower tumor weight in the GEM@PLGA@HM group compared to others. This confirmed the remarkable anti-cancer activity of GEM@PLGA@HM, attributed to its HER2 antibody-directed tumor targeting, enhanced accumulation at HER2^+^ tumor sites, and the biomimetic advantage of macrophage membrane camouflaging. Crucially, no appreciable alterations in body weight were discernible across any of the treatment groups (Fig. 7F). As shown in Fig. 7G and S11, the GEM@PLGA@HM treatment group provoked neither discernible pathological damage in vital organs such as the heart, liver, spleen, lungs, and kidneys, nor abnormal alterations in the levels of serum biomarkers, including ALT, AST, ALB, BUN, CR, and UA (*p* > 0.05). These experimental findings suggest that GEM@PLGA@HM exhibits negligible *in* *vivo* toxicity, favorable tumor-cell targeting capabilities and excellent biocompatibility.

Furthermore, we undertook a detailed mRNA expression analysis utilizing quantitative reverse transcription PCR (RT-qPCR) targeting seven crucial genes: *PI3K, AKT, MAPK,* *p53, Caspase-3, CCND1,* and *Ki67*, which are fundamentally involved in tumor proliferation, apoptosis, and cell cycle regulation. The outcomes presented in Fig. 7H–N correspond well with the cellular-level gene expression changes depicted in Fig. 5. Notably, the expression of genes within the PI3K/AKT signaling pathway (Fig. 7H and I) underwent marked suppression in response to the HER2-scFv presented on the macrophage membrane, particularly when formulated with PLGA@HM or GEM@PLGA@HM. Additionally, MAPK levels showed a decrease in tumors treated with GEM@PLGA, GEM@PLGA@M, and GEM@PLGA@HM (Fig. 7J). This synergistic action resulted in a notable upregulation of *p53* levels in the tumor tissues of the GEM@PLGA@HM-treated group (Fig. 7K). Consequently, this led to a downregulation of *CCND1*, a cyclin that plays a key role in cell cycle progression (Fig. 7M), and *Ki67*, a proliferation-related marker (Fig. 7N). Concurrently, there was an enhanced expression of *Caspase-3*, a crucial factor in the apoptotic process (Fig. 7L). In the subcutaneous breast tumor model, immunohistochemical staining also revealed that GEM@PLGA@HM markedly reduced *PI3K*, *AKT*, and *MAPK* expression and elevated *p53* expression when compared to PLGA@HM (Fig. S12). These observations were in excellent agreement with the aforementioned RT-qPCR results, indicating that GEM@PLGA@HM inhibits tumor cell proliferation and promotes apoptosis via dual modulation of PI3K/AKT and MAPK pathways, and thereby exerting anti-tumor effects.

These findings underscore the robust targeted therapeutic efficacy of GEM@PLGA@HM against HER2-positive tumors in mice. This therapeutic strategy works by intervening in multiple cellular signaling pathways through cytokines, ultimately activating the p53 pathway and promoting tumor cell apoptosis. These compelling results provide strong *in* *vivo* evidence supporting the therapeutic potential of GEM@PLGA@HM.

### 3.7 Programmed anti-HER2^+^ tumor metastasis of GEM@PLGA@HM *invivo*

**Fig. 8:**
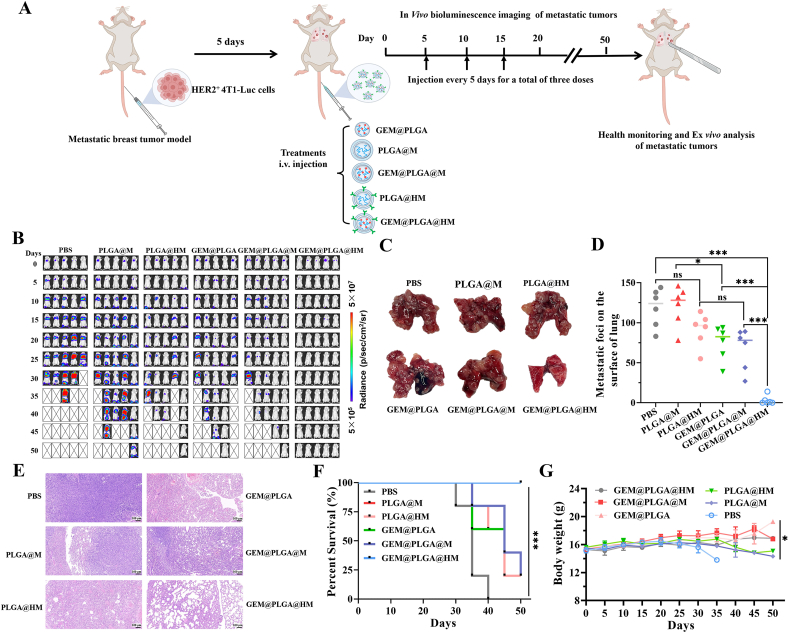
*Invivo* anti-metastasis effect of GEM@PLGA@HM for HER2 positive 4T1 tumor. (A) Schematic illustration of treatment schedule, *invivo* imaging and *exvivo* analysis using a metastatic breast tumor model. (B) Bioluminescence of tumor tissue of HER2 positive 4T1-Luc bearing mice intravenously injected with PBS, PLGA@M, GEM@PLGA, GEM@PLGA@M, PLGA@HM and GEM@PLGA@HM respectively in the metastatic tumor experiment models (n = 5). (C) The metastatic lesions of dissected lung tissues, and (D) statistics of lung metastatic foci for mice in different treatment groups in HER2 positive 4T1 models. (E) H&E staining sections of lung tissues of different treatment groups. Scale bar = 100 μm. (F, G) *Invivo* safety evaluation of different nano-carriers in metastatic HER2 positive 4T1 tumor models. (F) Survival curve and (G) changes in body weight of mice in different treatment days. The data are presented as means ± SD. ∗, ∗∗, and ∗∗∗ represent *p* < 0.05, *p* < 0.01, and *p* < 0.001, respectively.

HER2 positive 4T1-Luc mouse model was utilized to evaluate the anti-metastasis effect of GEM@PLGA@HM *in* *vivo*. The animals were divided in to six groups: (I) control (administered PBS 200 μL); (II) PLGA@M; (III) PLGA@HM; and (IV) GEM@PLGA; (Ⅴ) GEM@PLGA@M; (Ⅵ) GEM@PLGA@HM. The nano-carriers were delivered intravenously to the model mice 3 times (n = 6 per group) at 5, 10 and 15 days after HER2^+^ 4T1-Luc cells inoculation, the bioluminescence of HER2^+^ 4T1-Luc tumor was monitored to track the growth and development of metastatic tumors *in* *vivo* (Fig. 8A). After 50 days of treatment, it was clearly observed that injected HER2^+^ 4T1-Luc cells accumulated mainly in the lung tissue and lung metastatic tumor foci were formed (Fig. 8B and C). During the whole monitoring process, the bioluminescence of mice treated with GEM@PLGA@HM was found to be markedly weakened, indicating the shrinking tumor tissues. Especially after 15 days, no obvious bioluminescence signal was detected in all mice of GEM@PLGA@HM treated group. In contrast, mice in the PBS group experienced rapid tumor development, a significantly higher rate of lung metastasis than the other groups, and even the occurrence of death on the 30th day. In the PLGA@M, GEM@PLGA and GEM@PLGA@M groups, although lung metastasis was alleviated to a certain extent in the early stage, there were still many mice suffered tumor recurrence and died eventually (Fig. 8B). In conclusion, GEM@PLGA@HM was demonstrated the optimal therapeutic effect in the present study, which could effectively inhibit and kill tumor cells for a long time, indicating the sustained and efficient anti-tumor lung metastasis capacity of GEM@PLGA@HM. The mice were subsequently dissected to examine their lung tissue. Tumor metastases in the lung tissue were happened in almost all the experimental mice, however, after receiving the combined therapy of anti-HER2 antibodies and GEM, the lung metastatic lesions of mice in GEM@PLGA@HM group were significantly reduced compared with other groups (Fig. 8C), which was confirmed by the statistical data of lung metastatic foci shown in Fig. 8D. Taken together, these results clearly demonstrated that GEM@PLGA@HM played a crucial role in enhancing the combined therapeutic efficacy of lung tumor metastases, illustrating that GEM@PLGA@HM could effectively inhibit tumor metastasis by rapidly and accurately targeting and killing tumor cells.

The lung tissues of the metastatic tumor model mice were subsequently made sections for H&E staining. As shown in Fig. 8E, the lung tissues of the PBS and the PLGA@M groups were very dense with rare alveoli, indicating that the existence of a large part of tumors. A small fraction of alveoli and large area of dense tumor tissue were observed in GEM@PLGA, GEM@PLGA@M and PLGA@HM treated groups. However, the staining result of the GEM@PLGA@HM group showed abundant and clear alveoli while tumor tissues were almost disappeared, indicating that GEM@PLGA@HM exhibited excellent inhibitory effect on lung tumor metastasis. Therefore, tumor metastasis was successfully reduced and suppressed by GEM@PLGA@HM through combined therapy.

Changes in the body weight and survival of mice were further recorded to estimate whether metastatic tumors affected the health of mice. Fig. 8F showed the survival curve of mice treated with PBS, PLGA@M, GEM@PLGA, GEM@PLGA@M, PLGA@HM and GEM@PLGA@HM for 50 days. Compared with other treatment, the GEM@PLGA@HM could not only efficiently prevent tumor metastasis, but also significantly improve the survival time and rate of mice. No mice in the GEM@PLGA@HM group were observed to die during the 50 days treatment. In contrast, all the mice in the PBS group died within 40 days. There were similar survival rates in the PLGA@HM, GEM@PLGA and GEM@PLGA@M groups, with about 9-11 of the 55 mice dying, indicating that monotherapy could only achieve limited therapeutic efficacy, mortality of mice would still occur with the extension of therapeutic time.

During the whole therapy process, no significant weight gain or loss was occurred in the mice treated with GEM@PLGA@HM. On the 25th day after establishment of the metastatic tumor model, mice in other groups showed different degrees of change in body weight, especially in PBS and PLGA@M groups, the body weight of mice were continuously decreased (Fig. 8G). The combined therapy of GEM@PLGA@HM could remarkably prolong the survival of mice, confirming the excellent biosafety of GEM@PLGA@HM.

## 4 Discussion

In recent years, cell membrane coating technology has attracted considerable attention in the field of nanomedicine [11,24]. As a natural biological material, cell membrane owns unique biological characteristics, such as good biocompatibility, precise targeting, ability to evade immune surveillance, and strong drug loading capacity. Virtually any type of cell membrane, from red blood cells (RBC) to cancer cells, can be coated onto the surface of NPs [25], resulting in nanomaterials with enhancing effects that can be customized for specific applications [26]. Cell-membrane-coated NPs (CMCNPs) have been shown to be effective drug delivery systems due to their extended cycle time and disease homing ability [[25], [26], [27]]. This membrane-derived nanomedical delivery system not only contributes to avoiding immune clearance, but also endows NPs with various cellular and functional mimicries [28]. The targeting ability of these biomimetic NPs is often mediated by proteins expressed on the source cells, which enables the NPs to interact specifically with various disease substrates. For example, the strategy of RBC membrane coating drugs was adopted to transport neuroprotective agent NR2B9C through the blood-brain barrier, and stroke homing peptide (SHp) was inserted into the RBC membrane to drastically prolong the systemic circulation of NR2B9C, enhance the active targeting of the ischemic area in the MCAO rats, and reduce ischemic brain damage [29]. Furthermore, neuroinflammation response which occurs in cerebral ischemia is typically characterized by the recruitment of leukocytes to lesions in the brain. Researchers took advantage of this unique opportunity to develop a drug delivery system, which includes cross-linked dendrigraft poly-L-lysine (DGL) nanoparticles containing catalase and modified with endogenous tripeptide PGP, showing high affinity to neutrophils. Delivery of catalase to ischemic subregions and cerebral neurocytes of MCAO mice was achieved by these neutrophil-mediated nanoparticles and the therapeutic outcome of cerebral ischemia was greatly improved [30]. Through combining the biomimetic effects of cell membranes with functional synthetic nanomaterials, membrane-mediated nano delivery technology enables high drug loading capabilities, immune escape, and highly targeted therapy, paving the way for the development of novel drug delivery systems that target tumor cells.

Generally, the targeted effect of the drug directly affects the therapeutic effect. Anti-HER2-modified macrophage membranes were initially constructed in the present study. To produce HER2-scFV-LV, 293T cells were transfected with a plasmid encoding the HER2-scFV sequence. Next, macrophages expressing anti-HER2 antibody were generated by transfecting macrophages with HER2-scFV-LV. In order to determine the expression of GFP on the cell membrane, the GFP sequence was encoded into the plasmid sequence. After transfection, it was found that the HER2-scFV-GFP protein was produced on 293T cells and macrophages, indicating the transmembrane expression of HER2-scFV. Due to the importance of HER2 antibody in immune recognition for biotherapy, whether transmembrane expression of HER2-scFV could inherit its recognition ability was further assessed. In this study, HMC was developed by extracting anti-HER2-modified macrophage membranes. The material characterization results supported the successful construction of a biomimetic nanoplatform based on the GEM@PLGA nanoparticles and anti-HER2-modified macrophage. Subsequently, *in* *vitro* and *in* *vivo* experimental results showed that our biomimetic nanoplatform could efficiently deliver drugs to the tumor site, providing solid foundation for deeper tumor-specific targeted therapy. In addition, by modifying the biomimetic properties of cell membranes through genetic engineering, traditional nanomaterials were endowed with more diversified functions, which helps to overcome their shortcomings such as poor stability and non-specific tissue accumulation, providing new possibilities for the development of more accurate and efficient therapeutic means.

With the continuous in-depth study of functional CMCNPs, the demand for cell membrane derived nanoparticles also tends to be more specific and detailed. Cell membranes used for CMCNPs are undergoing a transition from natural to modified. At the same time, engineering strategies have expanded from initial encapsulation to lipid insertion, membrane hybridization, metabolic engineering, and genetic modification [31], contributing multiple functions in a non-disruptive way while preserving the natural function of the cell membranes. These approaches also improve on the multifunctional and multitasking capabilities of cell membrane-coated nanoparticles, making them more adaptable to the complexity of biological systems. In breast cancer research, chemotherapy remains one of the main therapeutic options for patients with poor prognostic factors. Over the past few decades, the prognosis of breast cancer patients has been significantly improved by chemotherapy with cyclophosphamide, methotrexate, and fluorouracil (CMF), followed by the introduction of anthracyclines and taxanes [32]. In the treatment of metastatic breast cancer, the addition of gemcitabine to taxanes resulted in obvious improvements in overall survival (OS) of patients, time to progression (TTP) and response rate (RR) [33]. Gemcitabine is a promising candidate for improving adjuvant cytotoxic therapy, which has shown excellent efficacy as a first-line treatment for metastatic cancer [[33], [34], [35], [36]]. However, off-target toxicity of small molecule drugs can lead to dangerous side effects, being a major cause of clinical trial failure. Therefore, the biomimetic nanoplatform designed in the present study utilizes anti-HER2 antibodies on the macrophage membrane to specifically recognize HER2 antigen on the tumor cells surface, achieving accurate GEM delivery and precise targeted therapy. The concept of the engineered anti-HER2 macrophage-drug conjugate (HMC) strategy and its anti-tumor effect were validated, which provide an inherent tumor-oriented tendency and intrinsic biotherapeutic function for effective targeting and therapy of tumors. Since the therapeutic effect of ADC drug payload is insignificant, the selection of GEM and PLGA nanocarriers as new adjuvant tools can create biomimetic nanoplatforms for HMC strategies, exhibiting synergistic anti-tumor effects *in* *vitro* and *in* *vivo*. After administration to HER2^+^ tumor-bearing mice, anti-HER2 engineered macrophage biomimetic photothermal nanoparticles (GEM@PLGA@HM) escaped the clearance of the macrophage system and actively targeted HER2-expressing tumor cells, resulting in increased accumulation of GEM@PLGA@HM in the tumor with minimal systemic toxicity. Due to the properties of PLGA, drug GEM can be successively released at tumor sites, thus destroying tumor cells and enhancing the anti-tumor effects of biologic therapies. GEM@PLGA@HM were further demonstrated to induce apoptosis of HER2^+^ tumor cells since the combined therapy intervenes in cell signaling pathways through activation of the apoptosis-related genes such as *p53*, indicating the strong targeted therapeutic effect of GEM@PLGA@HM on HER2-positive tumors in mice. In summary, the anti-HER2 macrophage membrane biomimetic nanoplatform was successfully constructed and applied to guide the implementation of synergistic chemotherapy and biotherapy, showing the desired anti-tumor efficacy. In addition to the above advantages, these HMC strategies can be extended to other antibodies and therapy, such as PD-L1, chemotherapy or radiotherapy. These characteristics are expected to bring great clinical translational potential for the treatment of breast cancer. The progress of biomimetic nanotechnology will promote the continuous development and innovation in the field of tumor chemotherapy.

Through the combination of engineered macrophage membrane and drug-loading nano particles, the strengths of both were taken and weaknesses were made up to achieve precise combined therapy, which not only relieved the adverse effects of GEM and improved its systemic circulation time *in* *vivo*, but also addressed the insufficient efficacy of antibodies monotherapy for tumors. In our study, the treatment of tumor-bearing mice also extended to the inhibition of metastatic lesions, which reflected the anti-metastasis ability of GEM@PLGA@HM, highlighting the precision targeting performance of the nano-system. In follow-up studies, the anti-metastasis effect of the system can be extended to transform therapy into obstruction, which may bring new insights into the field of biomimetic nanocarriers. Although the engineered cell membrane biomimetic nano-delivery system shows its tremendous innovation in drug delivery and has made remarkable progress in the field of tumor precise targeted therapy, however, there are still some problems in this field that need to be further explored and discussed. For example, more targets can be screened and multiple pathways be combined to further improve the precise therapy. By changing the combination of chemotherapy drugs or antibodies, the killing treatment of metastatic lesions will be transformed into blocking the metastasis pathway, so that the biomimetic drug loading nanoparticles could affect the generation and development of metastatic lesions much earlier.

## 5 Conclusion

Due to the unique abilities of biomimicry and biointerfacing, cell membrane-derived nanoparticles provide a new therapeutic strategy for the development of nano drug-delivery system with high-targeting and low-immunogenicity. In the present study, gene editing and camouflaging of HER2^+^ antibody-armed macrophage membranes were utilized to develop a novel targetable biomimetic nanoplatform for targeted delivery of anti-tumor drug GEM, thus achieving the combined therapeutic effect of the HER2^+^ antibody mediated biotherapy and chemotherapy against HER2^+^ tumors. Compared with unmodified macrophage membrane-coated nanocarriers, the GEM@PLGA@HM was demonstrated excellent HER2-targeting ability *in* *vitro* and *in* *vivo* and showed significantly increased GEM enrichment at HER2^+^ cancer cells and tumor sites. GEM@PLGA@HM induced enhanced cytotoxicity to HER2^+^ cancer cells by combination of the apoptosis effect induced by GEM and the blocking of the HER2 pathway, thus suppressing tumor growth and metastasis of HER2^+^ tumor-bearing mice. This study provides new strategies of a novel biomimetic nanoplatform designed for future clinical therapy of metastatic cancers.

## CRediT authorship contribution statement

**Xinan Wang:** Writing – original draft, Investigation, Formal analysis. **Wenhui Chu:** Data curation, Software, Validation, Writing – original draft. **Fuyu Du:** Writing – original draft, Software, Data curation. **Hongyu Fan:** Methodology, Formal analysis, Data curation. **Zixuan Ye:** Validation, Methodology. **Yaru Xue:** Writing – original draft. **Xianghan Zhang:** Validation, Methodology. **Peng Yang:** Validation, Methodology. **Yuqiong Xia:** Validation, Methodology. **Zhihui Chen:** Supervision, Resources, Project administration, Funding acquisition, Conceptualization. **Pengbo Ning:** Writing – review & editing, Validation, Supervision, Project administration, Investigation, Funding acquisition, Formal analysis, Data curation, Conceptualization.

## Declaration of competing interest

The authors declare that they have no known competing financial interests or personal relationships that could have appeared to influence the work reported in this paper.

## Data Availability

Data will be made available on request.
