# Supplementary material for: Synergistic HER2-scFv mediated immunotherapy with targeted gemcitabine delivery harnessing biomimetic composite nanoparticles for enhanced metastatic tumor therapy
Source: Mater Today Bio. 2025 Apr 22;32:101784. doi: 10.1016/j.mtbio.2025.101784 (PMC12423602; doi:10.1016/j.mtbio.2025.101784)
Supplement: Multimedia component 1 [file mmc1.docx]

**Synergistic HER2-scFv mediated immunotherapy with Targeted Gemcitabine Delivery Harnessing Biomimetic Composite Nanoparticles for Enhanced Metastatic Tumor Therapy**

Authors

Xinan Wang^1,2†^, Wenhui Chu^1,2†^, Fuyu Du^1,2^, Hongyu Fan^1,2^, Zixuan Ye^1,2^, Yaru Xue^1,2^, Xianghan Zhang^1,2^, Peng Yang^1,2^, Yuqiong Xia^1,2^, Zhihui Chen^3, 4*^, Pengbo Ning^1,2*^

Affiliations

^1^School of Life Science and Technology, Xidian University, Xi’an, Shaanxi 710071, PR China;

^2^Engineering Research Center of Molecular & Neuroimaging, Ministry of Education, Xi’an, Shaanxi 710071, PR China;

^3^Department of Gastrointestinal Surgery Center, The First Affiliated Hospital, Sun Yat-sen University, Guangzhou 510080, PR China;

^4^Department of General Surgery, Guangxi Hospital Division of The First Affiliated Hospital, Sun Yat-sen University, Nanning, Guangxi 530022, PR China.

*Correspondence to: Zhihui Chen ([chzhhui@mail.sysu.edu.cn](mailto:chzhhui@mail.sysu.edu.cn).); Pengbo Ning ([pbning@xidian.edu.cn](mailto:pbning@xidian.edu.cn)).

† These authors contributed equally to this work.

**Supplementary information**


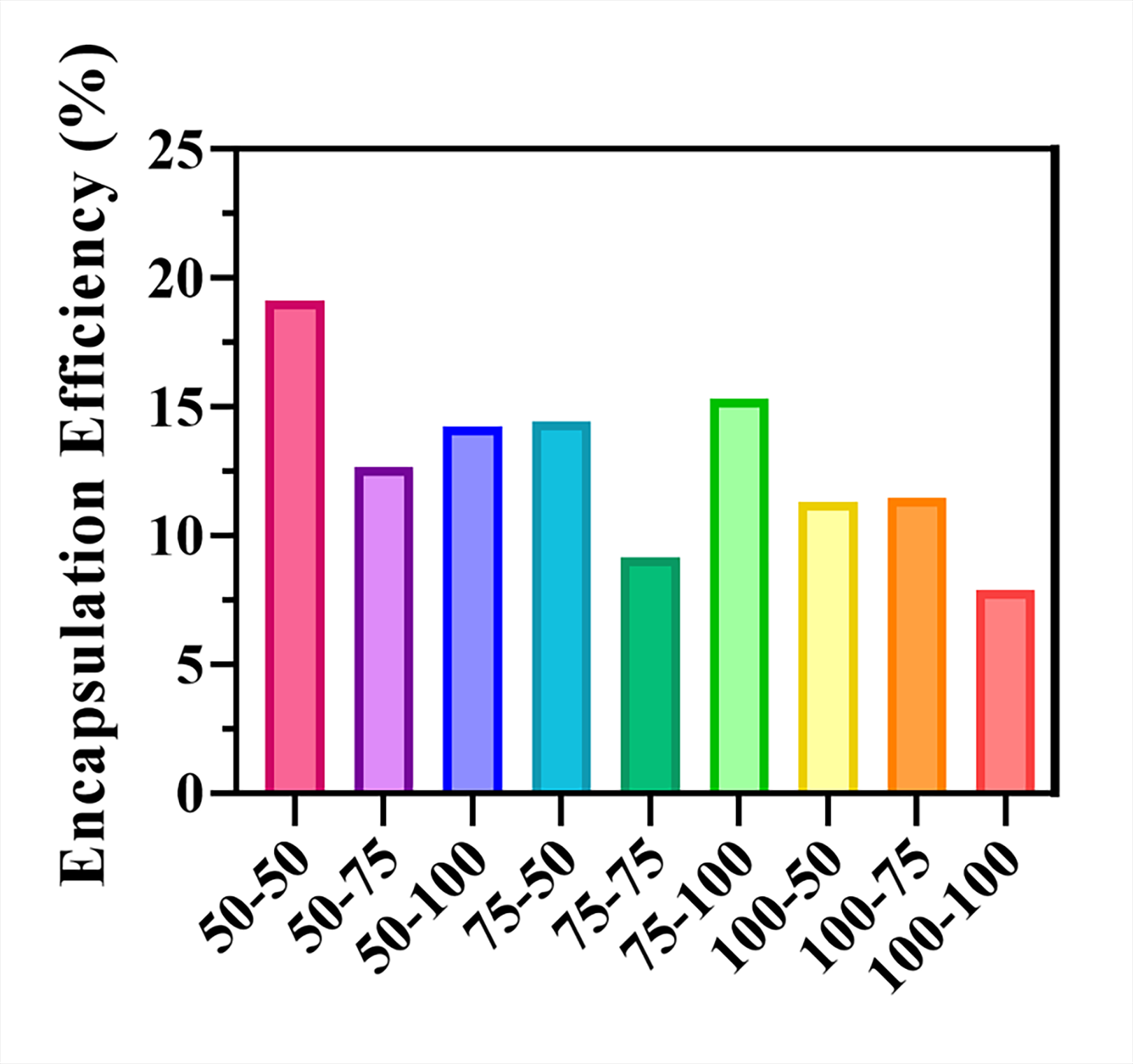


**Figure S1. Comparison of the encapsulation efficiency of PLGA nanoparticles under different ultrasonic intensities combination.**


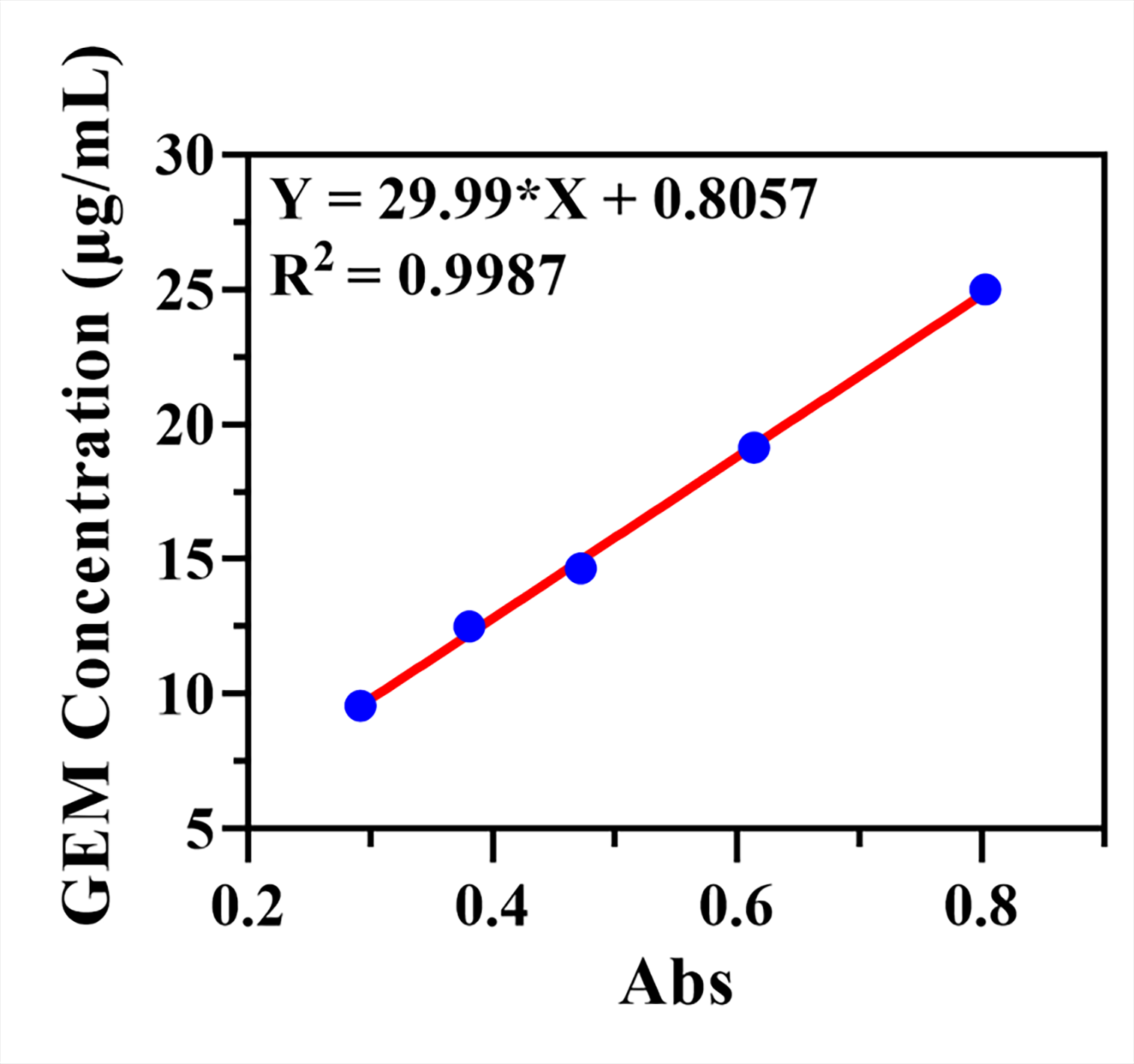


**Figure S2. The standard curve of GEM** **at 269 nm UV absorption.**


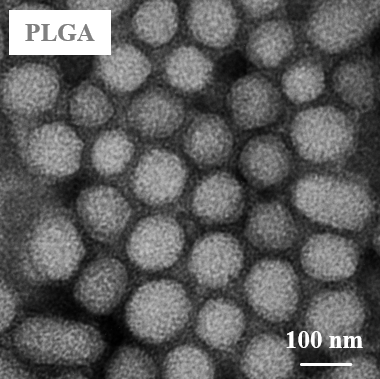


**Figure S3. Representative TEM image of PLGA NPs (scale bar, 100 nm).**

**
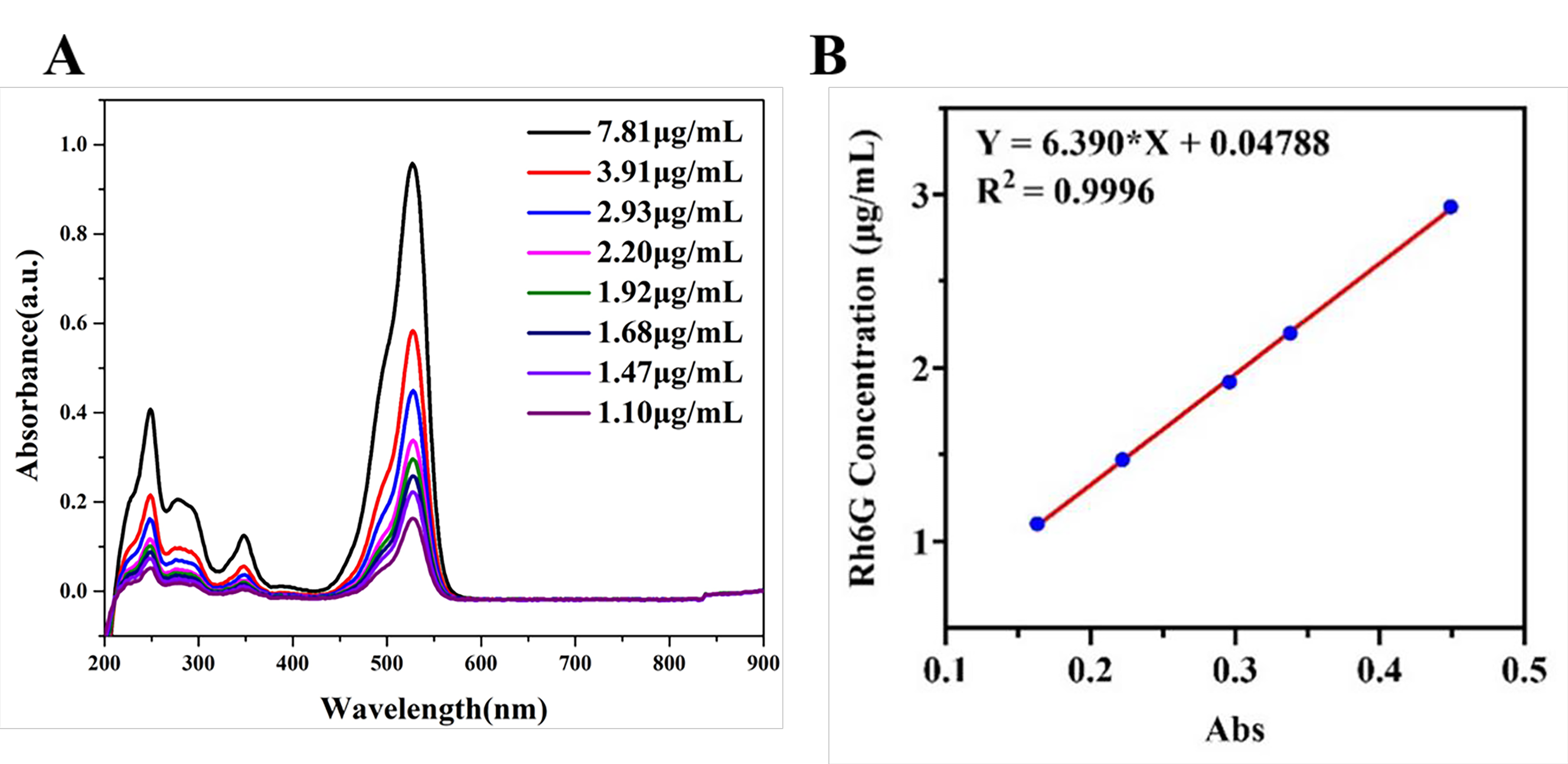
**

**Figure S4. (A) The ultraviolet−visible (UV) absorption spectra and (B) standard curves of rhodamine 6G (Rh6G) fluorescent dye at 530 nm.**

**
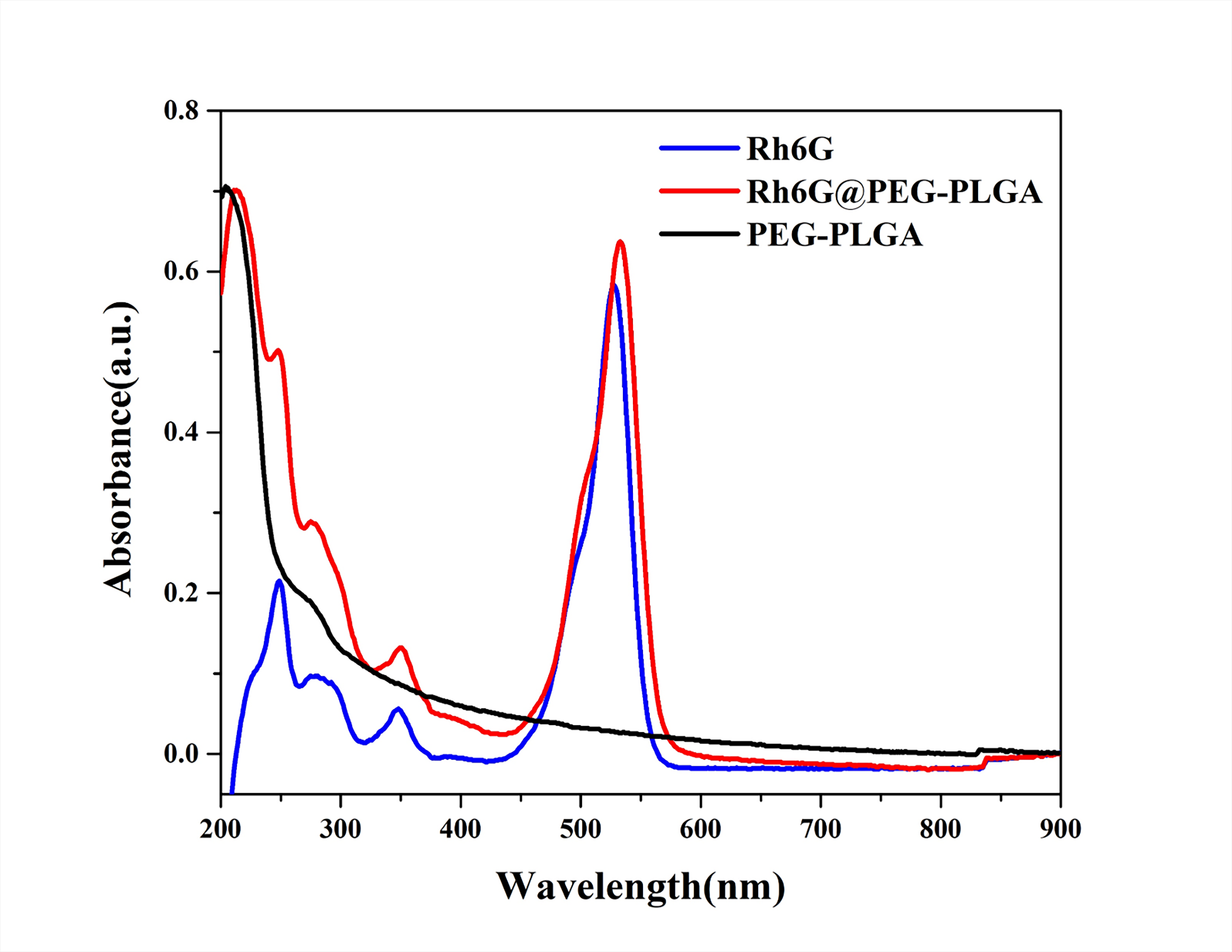
**

**Figure S5. The ultraviolet−visible (UV) absorption spectra of Rh6G, Rh6G@PEG-PLGA and PEG-PLGA NPs.**

**
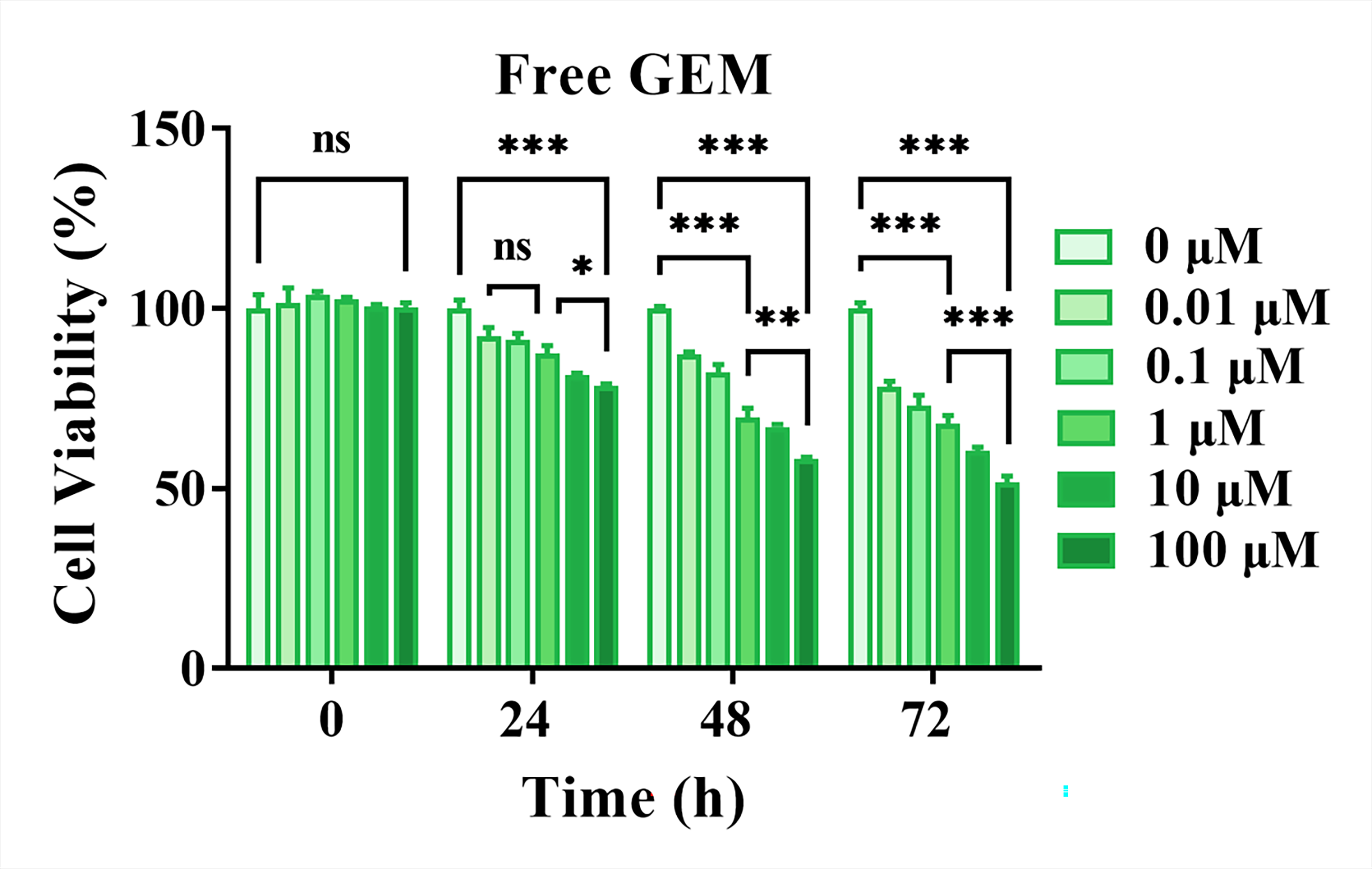
**

**Figure S6.** ***In vitro* safety evaluation of free GEM.** J774A.1 cells were incubated with different concentrations (0, 0.01, 0.1, 1, 10 and 100 μM/L) of free GEM for 0, 24, 48, and 72 h, then the cells viability was analyzed using the CCK-8 assay. The data are presented as means ± SD. *, **, and *** represent *p* < 0.05, *p* < 0.01, and *p* < 0.001, respectively.

**
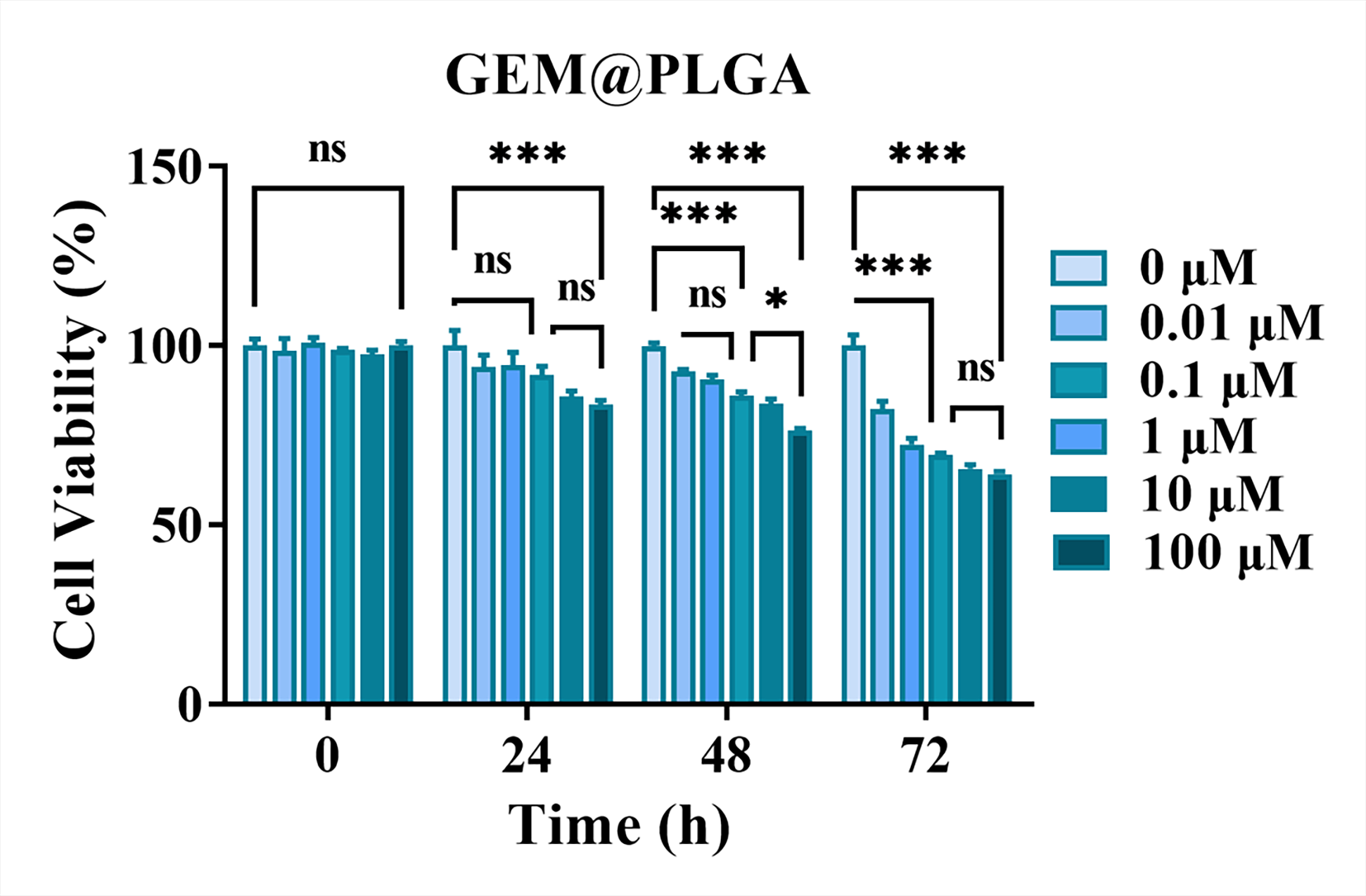
**

**Figure S7. *In* *vitro* safety evaluation** **of GEM@PLGA.** J774A.1 cells were incubated with different concentrations (0, 0.01, 0.1, 1, 10 and 100 μM/L) of GEM@PLGA for 0, 24, 48, and 72 h, then the cells viability was analyzed using the CCK-8 assay. The data are presented as means ± SD. *, **, and *** represent *p* < 0.05, *p* < 0.01, and *p* < 0.001, respectively.

**
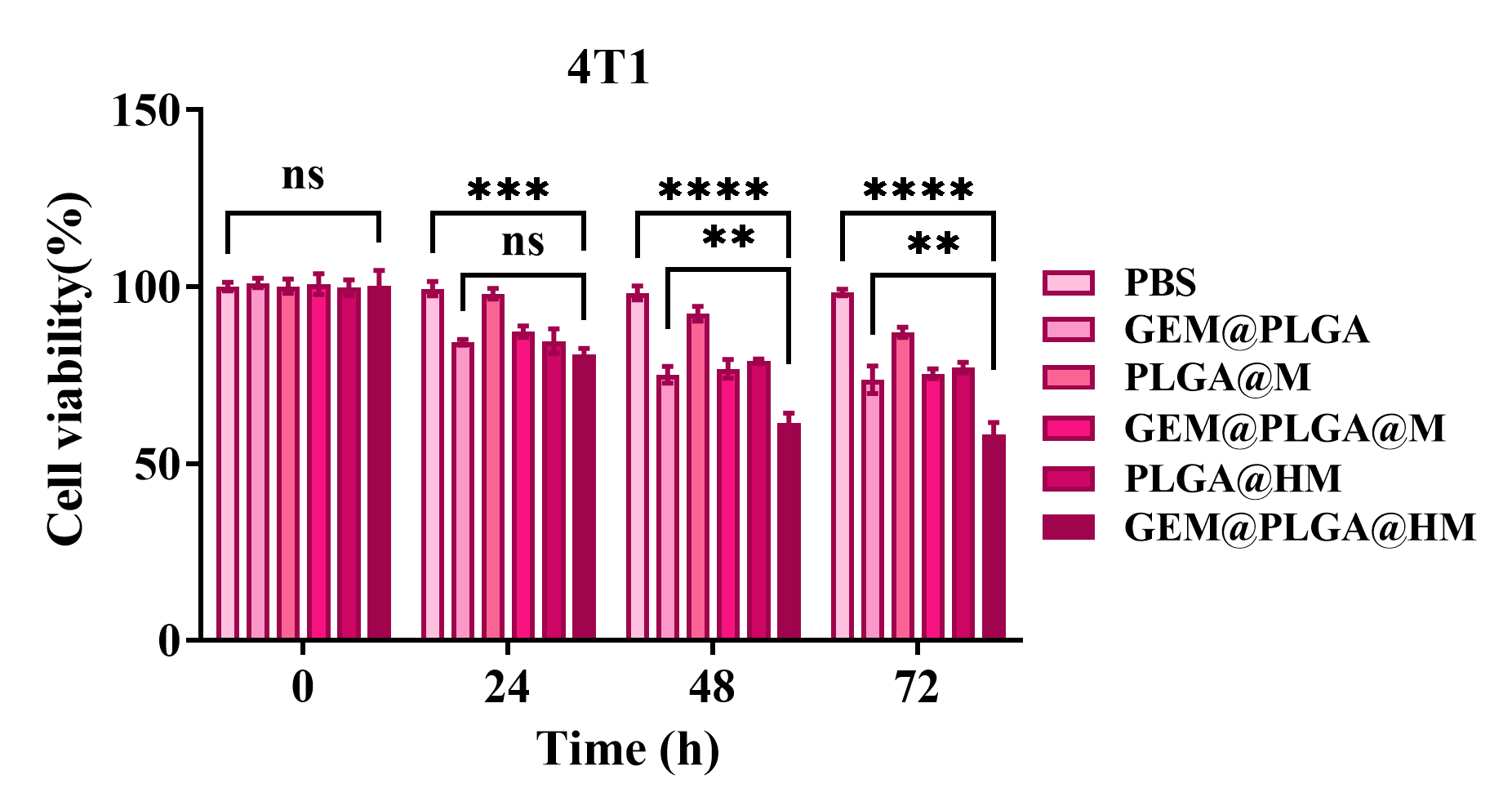
**

**Figure S8.** **Comparison of the *in* *vitro* time-gradient cytotoxicity of all PLGA nanocarriers to 4T1 cells**. HER2^+^ 4T1 cells were incubated with PBS, GEM@PLGA, PLGA@M, GEM@PLGA@M, PLGA@HM and GEM@PLGA@HM (GEM: 1 μM/L) for 0, 24, 48 and 72 h separately. **, ***and **** represent *p* < 0.01, *p* < 0.001, and *p* < 0.0001, respectively.

**
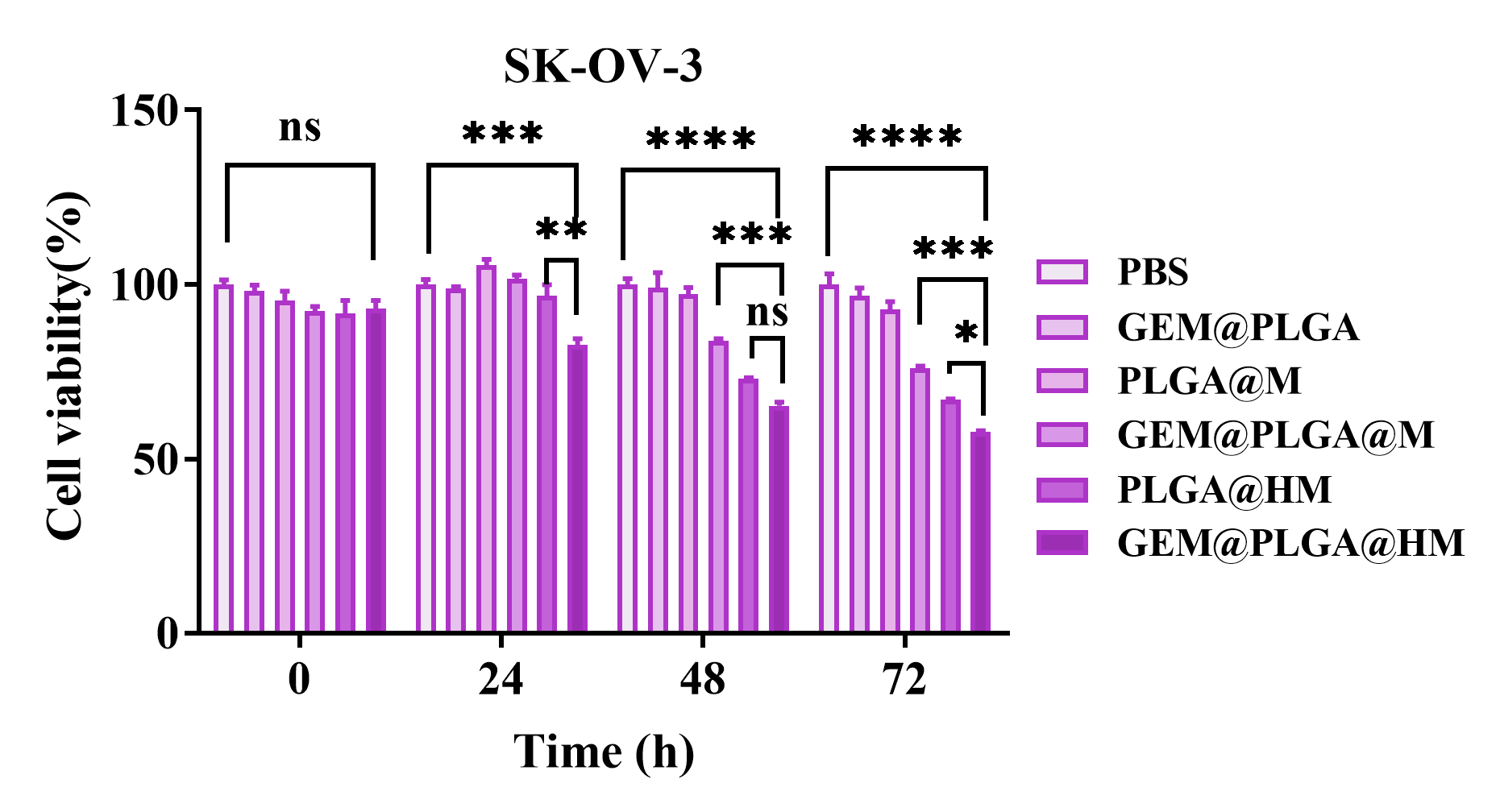
**

**Figure S9. Comparison of the *in vitro* time-gradient cytotoxicity of all PLGA** **nanocarriers to SK-OV-3 cells.** SK-OV-3 cells were incubated with PBS, GEM@PLGA, PLGA@M, GEM@PLGA@M, PLGA@HM and GEM@PLGA@HM (GEM: 1 μM/L) for 0, 24, 48 and 72 h separately. *, **, ***and **** represent *p* < 0.05, *p* < 0.01, *p* < 0.001, and *p* < 0.0001, respectively.

**
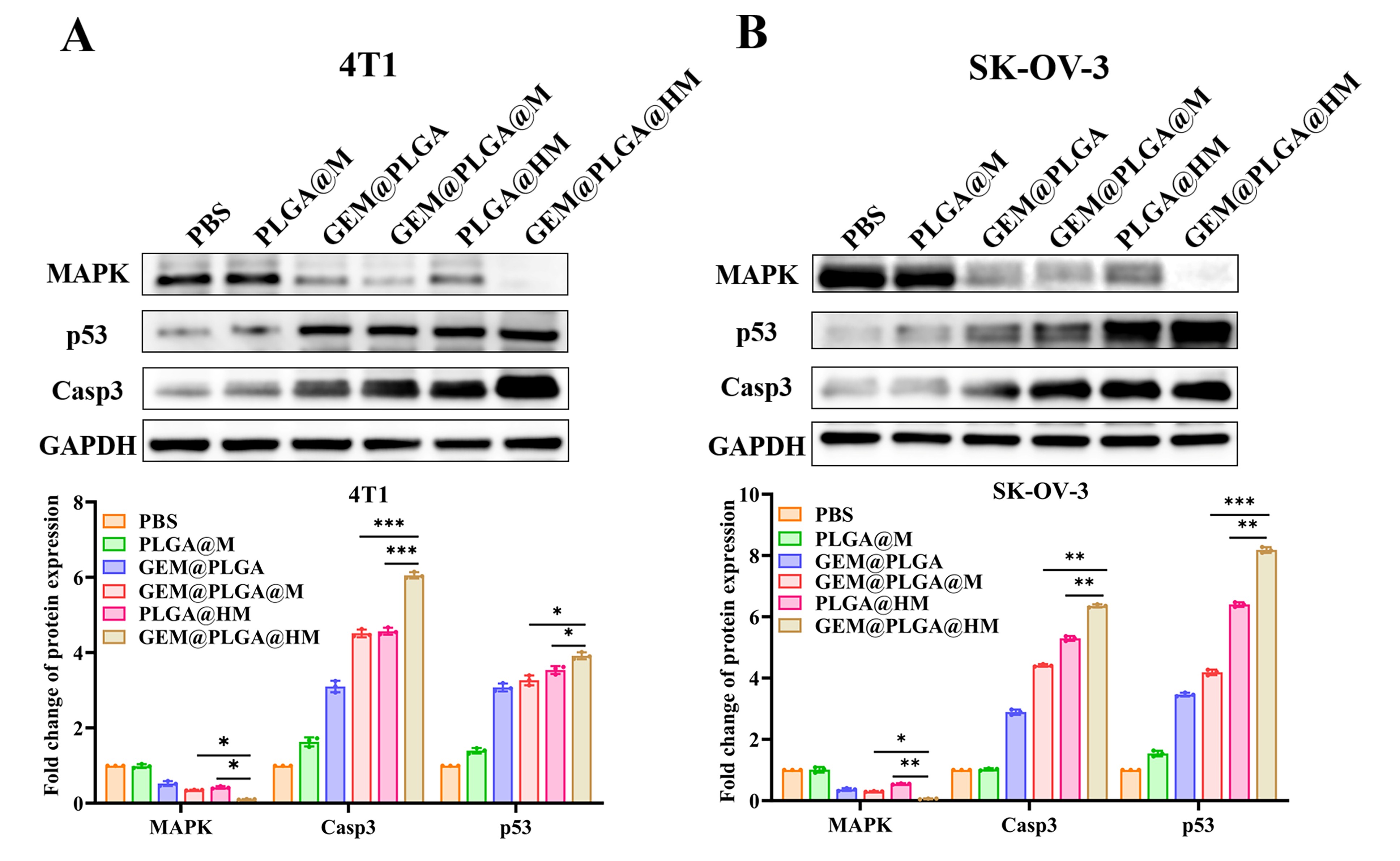
**

**Figure S10**. **Western blot analysis of key genes expression in HER2^+^ tumor cells after different treatments.** The expression of MAPK, p53 and Caspase-3 in (A) 4T1 cells and (B) SK-OV-3 cells treated with PBS, PLGA@M, GEM@PLGA, GEM@PLGA@M, PLGA@HM and GEM@PLGA@HM (GEM: 1 μM/L) for 24 h, respectively. The data are presented as means ± SD. *, **, and *** represent *p* < 0.05, *p* < 0.01, and *p* < 0.001, respectively.

**
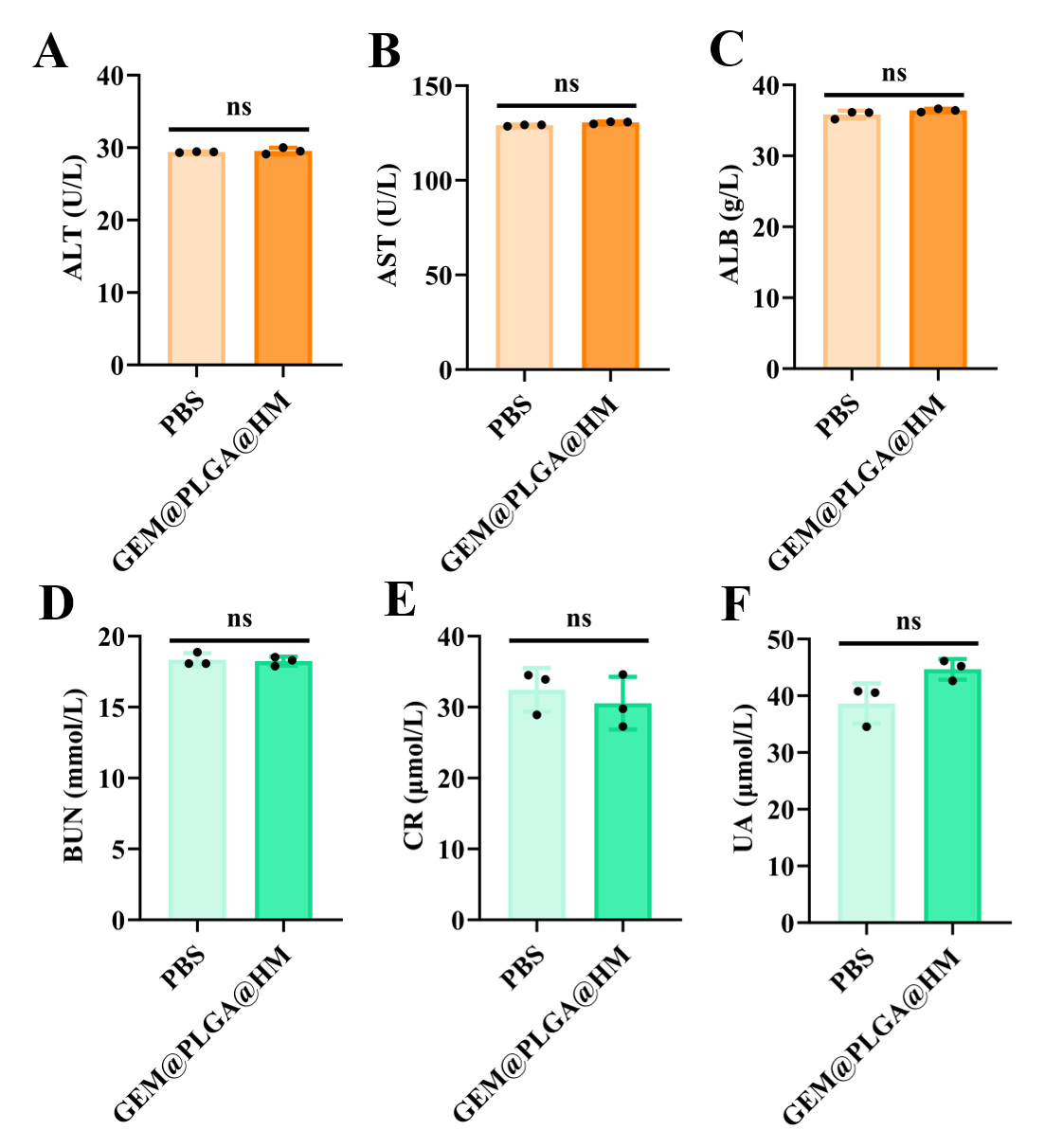
**

**Figure S11**. **Hematological analysis of the *in* *vivo* safety of GEM@PLGA@HM.** Blood samples were collected from mice treated with PBS or GEM@PLGA@HM and were utilized to measure the levels of (A) alanine aminotransferase (ALT), (B) aspartate aminotransferase (AST), (C) albumin (ALB), (D) blood urea nitrogen (BUN), (E) creatinine (CR), and (F) uric acid (UA).

The data are presented as means ± SD.

**
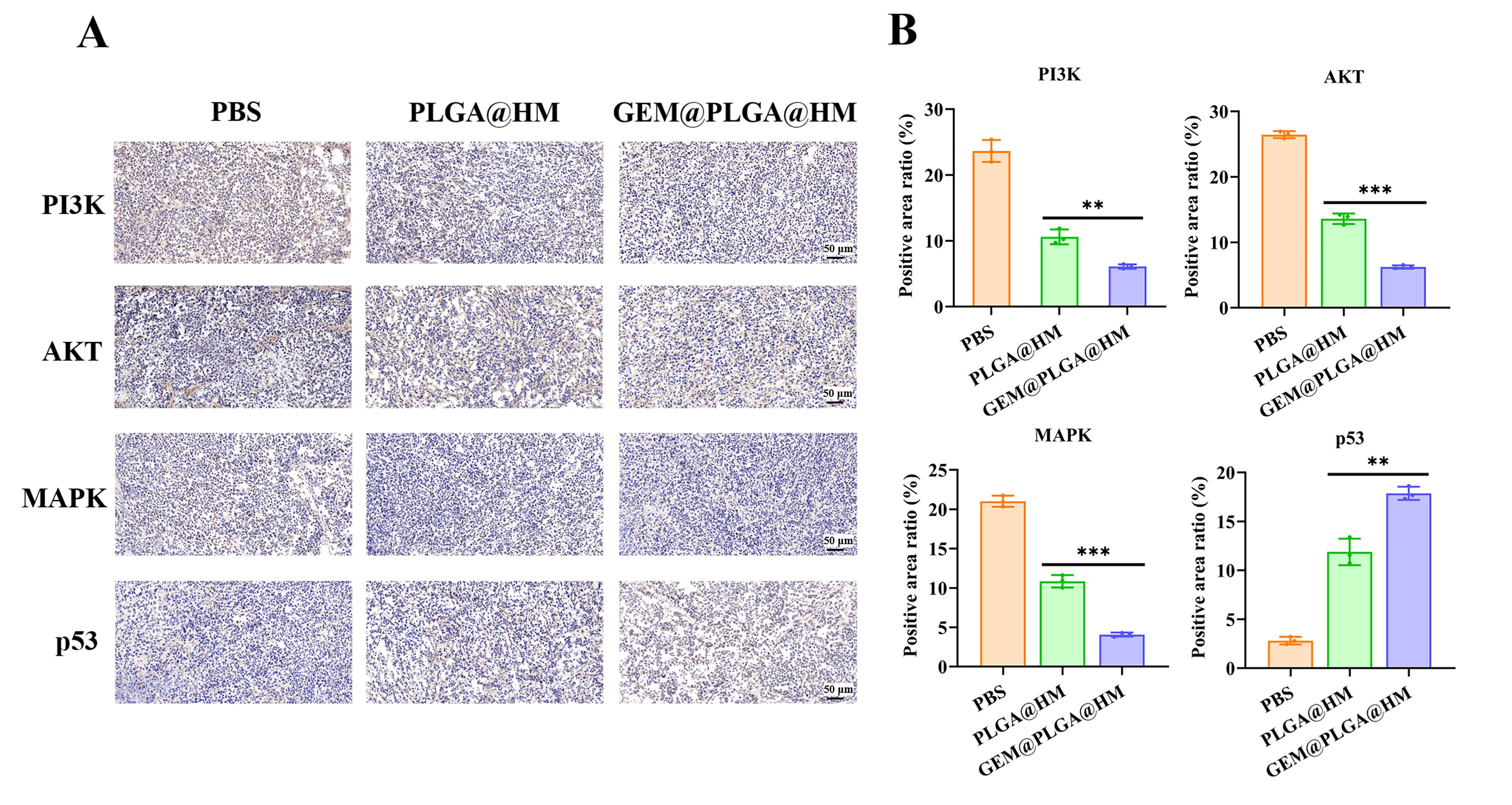
**

**Figure S12**. (A) Immunohistochemistry (IHC) staining results showing the expression profiles of P13K, AKT, MAPK, and p53 in tumor region slices derived from HER2^+^ tumors, following treatment with PBS, PLGA@HM, and GEM@PLGA@HM, respectively. Scale bar = 50 μm. (B) Quantification analysis presenting the expression levels of the specified genes. The data are presented as means ± SD. ** and *** represent *p* < 0.01 and *p* < 0.001, respectively.

**Table S1. Properties comparison of nanoparticles synthesized under different ultrasonic intensities combination.**

**
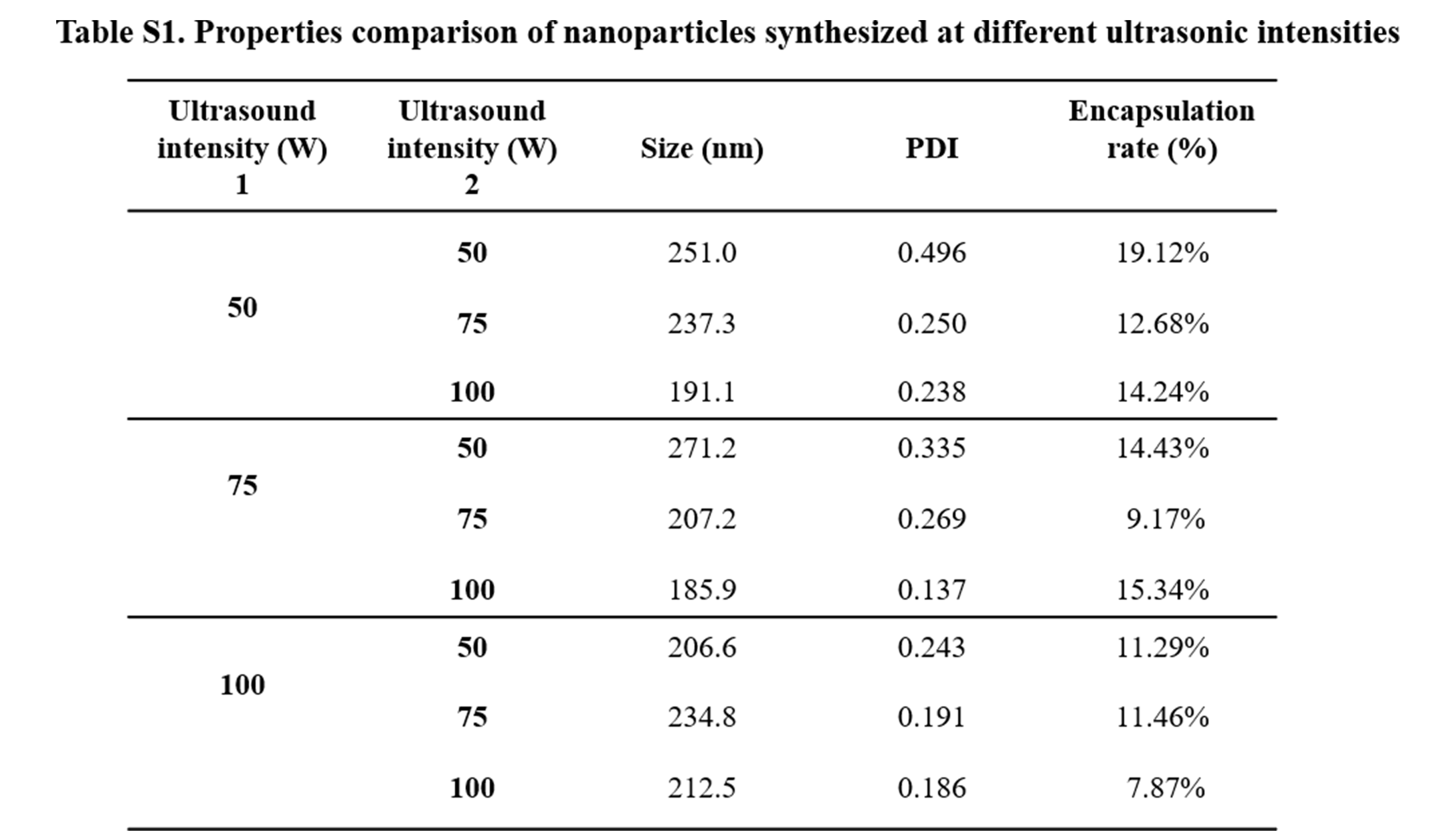
**

**Table S2. The mouse primer information for quantitative polymerase chain reaction (RT-qPCR).**

**
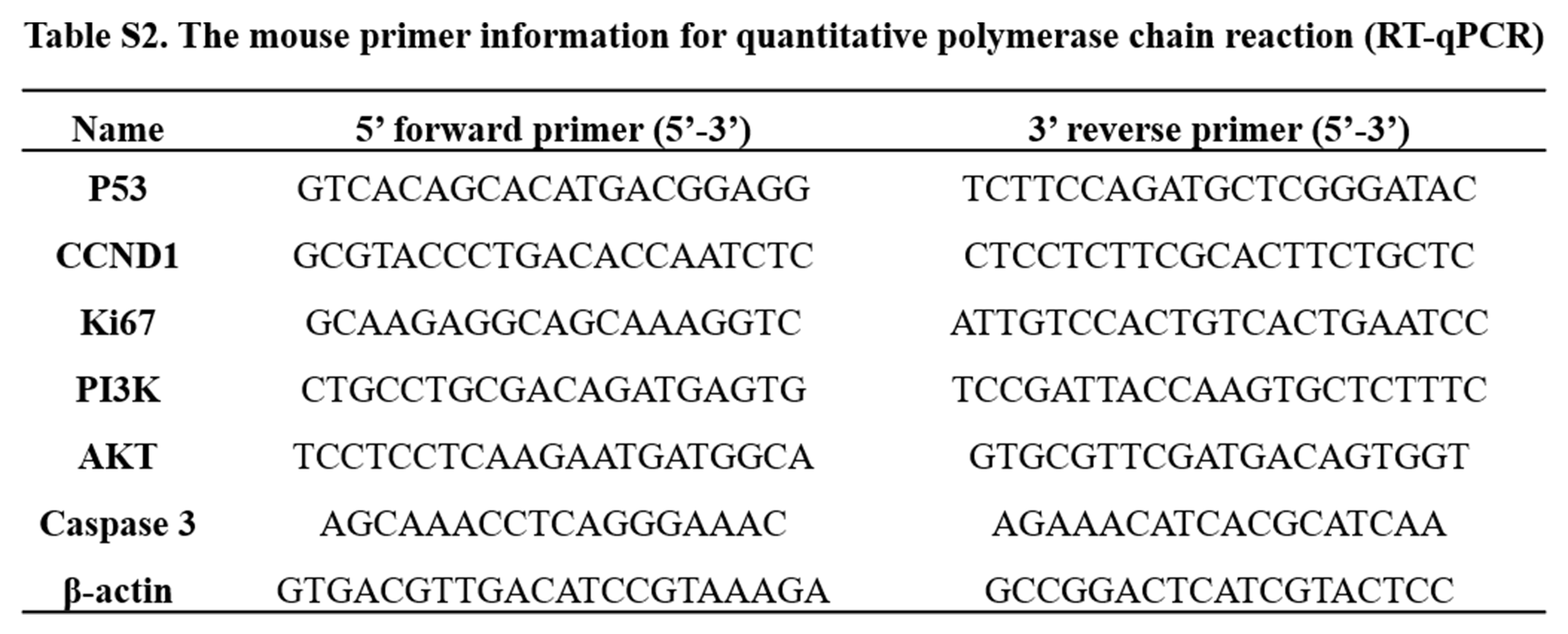
**

**Table S3. The human primer information for quantitative polymerase chain reaction (RT-qPCR)**

**
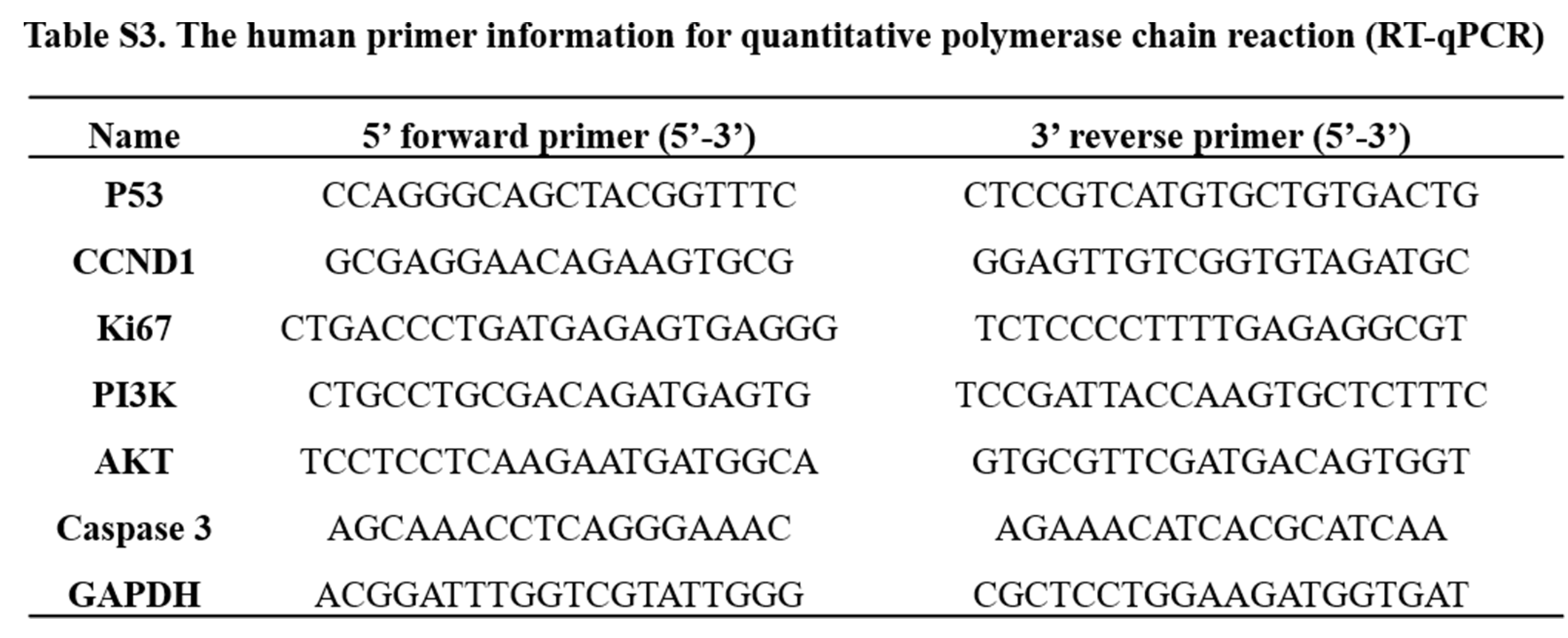
**
